# Perovskite Surface Treatment with Cu_2_O‑MIL-53 (Al) Metal-Organic Frameworks for Enhanced Efficiency and Stable Perovskite Solar Cells

**DOI:** 10.1021/acsomega.5c12575

**Published:** 2026-08-12

**Authors:** Sorrawit Meeklinhom, Madsakorn Towannang, Jirasak Gamonchuang, Kitiphat Sinthiptharakoon, Komsun Lapawae, Nuttaya Sukgorn, Nopporn Rujisamphan, Chalida Imhan, Chalita Ratanatawanate, Pipat Ruankham, Duangmanee Wongratanaphisan, Pongsakorn Kanjanaboos, Anusit Kaewprajak, Pisist Kumnorkaew

**Affiliations:** † National Nanotechnology Center (NANOTEC), 371383 National Science and Technology Development Agency (NSTDA), 111 Thailand Science Park, Pathum Thani 12120, Thailand; ‡ Thailand Center of Excellence in Physics (ThEP Center), 26682 Ministry of Higher Education, Science, Research and Innovation, Bangkok 10400, Thailand; § Nanoscience and Nanotechnology Graduate Program, Faculty of Science, 65128 King Mongkut’s University of Technology Thonburi, Bangkok 10140, Thailand; ∥ Department of Physics and Materials Science, Faculty of Science, Chiang Mai University, Chiang Mai 50200, Thailand; ⊥ School of Materials Science and Innovation, Faculty of Science, 26685 Mahidol University, Nakhon Pathom 73170, Thailand

## Abstract

Defect formation in perovskite films, particularly due to uncontrolled lead iodide residues, remains a critical challenge in perovskite solar cells (PSCs), leading to nonradiative recombination, voltage losses, and rapid device degradation. Surface passivation is therefore essential to suppress recombination and enhance device stability. In this work, we introduce a simple surface modification strategy using Cu_2_O–MIL-53­(Al) (CuM) nanoparticles, deposited by spin coating onto perovskite films. The incorporation of CuM effectively regulates residual PbI_2_, promotes the reduction of the relative PbI_2_-related diffraction contribution, and modifies the interfacial chemical environment. As a result, the optimized device achieved a power conversion efficiency (PCE) of 20.25% with an open-circuit voltage (*V*_oc_) of 1.11 V and negligible hysteresis. Photophysical and electrochemical analyses further indicate reduced nonradiative recombination and improved interfacial carrier dynamics after CuM treatment. In addition, unencapsulated devices retained 96.56% of their initial efficiency after 1000 h under ambient humidity, and the CuM-treated device also exhibited improved thermal-storage stability at 65 °C under N_2_. These results identify CuM surface treatment as an effective interfacial-engineering approach for improving the performance and storage stability of PSCs.

## Graphical abstract

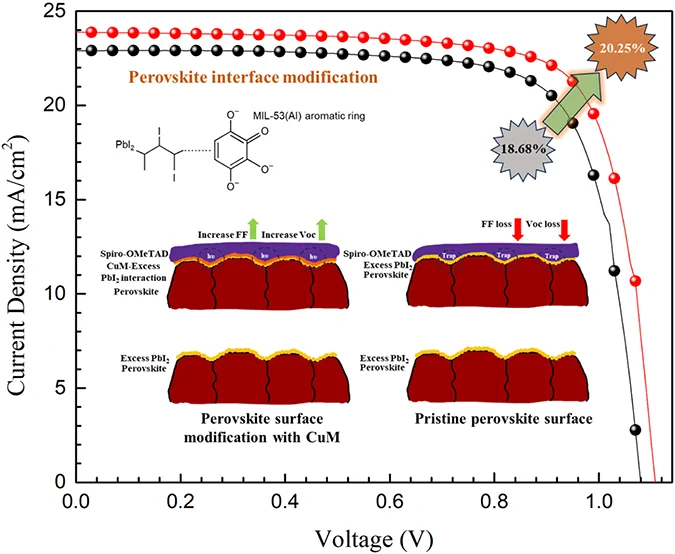

## 1 Introduction

Perovskite solar cells (PSCs) have rapidly emerged as one of the most promising photovoltaic technologies of the past decade, with certified power conversion efficiencies (PCEs) increasing from 3.8% in 2009 to over 25% today.^1,2^ This remarkable progress is attributed to the excellent optoelectronic properties of organic–inorganic halide perovskites, including high absorption coefficients, tunable bandgaps, long carrier diffusion lengths, and solution-processability.^3,4^ These unique features render PSCs attractive candidates for low-cost, high-efficiency photovoltaic devices. However, despite such rapid advancement, the long-term operational stability and interfacial losses in PSCs remain critical challenges that hinder large-scale commercialization.^5,6^

One of the primary bottlenecks arises from defects at the perovskite surface and grain boundaries. Nonstoichiometric phases, particularly residual PbI_2_, tend to accumulate at these interfaces, creating nonradiative recombination centers that reduce open-circuit voltage (*V*_oc_), limit charge extraction, and accelerate degradation.^7–9^ In addition, poor interfacial contact with charge transport layers further exacerbates recombination losses, leading to efficiency instability under operating conditions.^10,11^ As a result, strategies that mitigate interfacial defects and simultaneously improve charge-transfer dynamics are indispensable for advancing PSCs technology.^12,13^

Surface passivation has been widely employed to suppress defect-assisted recombination. Various molecular additives, polymers, quantum dots, and low-dimensional materials have been introduced to passivate dangling bonds, reduce trap densities, and enhance interfacial energy alignment.^14,15^ Among these strategies, metal–organic frameworks (MOFs) have attracted increasing attention due to their structural versatility, tunable porosity, and chemical functionality.^16,17^ MOFs can serve as multifunctional modifiers, offering large surface areas for defect passivation, π-conjugated linkers for charge delocalization, and chemical stability under processing conditions.^18,19^ Several studies have demonstrated the successful incorporation of MOFs as interlayers or additives in PSCs, resulting in improved perovskite crystallinity, defect reduction, and enhanced environmental stability.^20–24^

Alongside MOFs, copper-based materials have been recognized as promising alternatives to conventional organic hole-transport layers (HTLs). Materials such as CuSCN, CuI, and Cu_2_O offer high hole mobility, suitable valence band alignment, environmental compatibility, and low cost.^25,26^ In particular, cuprous oxide (Cu_2_O) has been highlighted for its excellent p-type conductivity and ability to facilitate hole extraction.^27,28^ However, the direct use of Cu_2_O in PSCs is limited by processing challenges: dissolution often requires toxic solvents such as diethyl sulfide (DES), which damage the perovskite layer, while vapor-phase deposition methods are energy-intensive and costly.^26,29,30^ Consequently, strategies that integrate Cu_2_O within a stabilizing framework are needed to harness its advantages while mitigating these limitations.

To this end, hybrid Cu_2_O–MOF composites have emerged as an innovative approach. By embedding Cu_2_O within a MOF matrix, it is possible to combine the efficient hole transport properties of Cu_2_O with the structural ordering, defect passivation, and interfacial stability offered by MOFs. Among available MOF structures, MIL-53­(Al), based on terephthalic acid (TPA) linkers and aluminum clusters, has been widely investigated due to its chemical stability, environmentally benign synthesis, and tunable electronic interactions.^16^ The aromatic rings of the TPA ligands provide conjugated π-electron systems capable of interacting with Pb^2+^ or I^–^ at the perovskite interface, stabilizing the surface and mitigating trap states.^15,21,22^ When combined with Cu_2_O nanoparticles, the resulting Cu_2_O–MIL-53­(Al) (CuM) composite represents a multifunctional interfacial modifier capable of enhancing both defect passivation and charge extraction.

Despite these promising attributes, the role of Cu_2_O–MIL-53­(Al) in PSCs remains underexplored. While previous studies have introduced MOFs and copper-based materials separately, there is still a lack of systematic understanding regarding how CuM influences PbI_2_-related defects reduction and modifies charge-transfer dynamics at the perovskite/HTL interface. Furthermore, the long-term operational stability of PSCs incorporating CuM surface modification has not been fully evaluated, particularly under ambient conditions without encapsulation. Addressing these gaps is crucial for advancing interfacial engineering strategies that are simple, scalable, and environmentally benign.

In this work, we report a facile surface passivation approach using Cu_2_O–MIL-53­(Al) nanoparticles (CuM) deposited via spin coating on perovskite films. This simple, low-cost strategy effectively controls PbI_2_ residues and improves interfacial contact between the perovskite and HTL. Optical and spectroscopic measurements demonstrate that CuM treatment suppresses nonradiative recombination without altering the perovskite bandgap, while time-resolved photoluminescence and electrochemical impedance spectroscopy reveal enhanced carrier lifetimes and reduced recombination resistance. As a result, PSCs incorporating CuM achieved a PCE of 20.25% with a *V*_oc_ of 1.11 V and negligible hysteresis. Remarkably, unencapsulated devices retained more than 96% of their initial efficiency after 1000 h under ambient humidity (40–60% RH), far outperforming pristine devices. These results establish CuM nanoparticle surface passivation as a promising pathway toward stable, high-efficiency PSCs and lay the groundwork for scalable interfacial engineering in future perovskite modules.

## 2 Materials and Methods

### 2.1 Materials

Cesium iodide (CsI, 99.9%), lead­(II) bromide (PbBr_2_, 99.999% trace metals basis), bis­(trifluoromethane)­sulfonimide lithium salt (Li-TFSI, 99.95%), 4-*tert*-butylpyridine (tBP, 96%), tris­(2-(1H-pyrazol-1-yl)­pyridine)­cobalt­(III) tris­(hexafluorophosphate) [FK102 Co­(III)­PF_6_, 98%], chlorobenzene (CB, anhydrous, 99.8%), N,N-dimethylformamide (DMF, anhydrous, 99.8%), dimethyl sulfoxide (DMSO, anhydrous, ≥99.9%), and methanol (MeOH) were purchased from Sigma-Aldrich (USA). Formamidinium iodide (FAI, 99.5%) and methylammonium bromide (MABr, >99.99%) were obtained from Greatcell Solar (Australia). Spiro-OMeTAD (99.9%) was purchased from Xi’an Yuri Solar Co., Ltd. (China). Ammonium chloride (NH_4_Cl, 98%) and SnO_2_ colloidal dispersion [tin­(IV) oxide, 15 wt % in H_2_O] were purchased from Alfa Aesar (USA). Lead­(II) iodide (PbI_2_, 99.99%) was obtained from TCI (Japan). 2-Butanol (99%), copper­(II) sulfate pentahydrate (CuSO_4_·5H_2_O, >98%), and sodium hydroxide (NaOH, 98%) were supplied by Carlo Erba (France). d-glucose was purchased from KEMAUS (Australia). Disodium terephthalate (Na_2_TP) was obtained via alkaline hydrolysis of PET. Aluminum nitrate nonahydrate [Al­(NO_3_)_3_·9H_2_O] was obtained from Jinan Boss Chemical Co., Ltd. (China).

### 2.2 Methods

#### 2.2.1 Synthesis of MIL-53­(Al)

MIL-53­(Al) was synthesized following a modified procedure based on a reported patent (Ratanatawanate et al., Thailand Patent TH 2201006356, 2022). A linker solution was prepared by dissolving 0.64 mol of Na_2_TP in 1.60 L of deionized (DI) water. Separately, 0.64 mol of Al­(NO_3_)_3_·9H_2_O was dissolved in 0.48 L of DI water to form the metal precursor solution. The two solutions were combined under overhead stirring 600 rpm at room temperature ∼23 °C for 5 min and then vacuum-filtered to yield a white powder. To activate the pores, the obtained solid was refluxed in 1 L of DMF at 130 °C for 6 h, followed by filtration, repeated washing with MeOH, and drying overnight at 70 °C. The final yield was 66.88 g of activated MIL-53­(Al).

#### 2.2.2 Preparation of Cu_2_O–MIL-53­(Al) (CuM)

CuM was synthesized by a modified literature method.^31^ Briefly, 1 g of MIL-53­(Al) was dispersed in 40 mL of DI water using ultrasonication and magnetic stirring for 30 min at ambient temperature. A 10% (w/v) aqueous solution of d-glucose was added dropwise under continuous stirring for 60 min. Subsequently, a 5% (w/v) CuSO_4_·5H_2_O solution was introduced dropwise over 10 min while heating at 85 °C. Thereafter, 10 mL of 1 M NaOH solution was added, and the mixture was stirred for an additional 60 min at the same temperature. The resulting CuM product was collected by centrifugation 12,000 rpm, washed repeatedly with DI water until neutral pH, and dried at 100 °C for 6 h. TEM images of the synthesized CuM composite, revealing that the Cu_2_O nanoparticles exhibited an average particle size of approximately 8–10 nm as shown in Figure S1.

#### 2.2.3 Device Fabrication

Prepatterned indium tin oxide (ITO) glass substrates (2.5 × 2.5 cm^2^, 6–8 Ω/sq) were sequentially cleaned by ultrasonication in detergent, DI water, acetone, and isopropanol (30 min each), followed by drying under compressed air. A 5 wt % SnO_2_ colloidal solution was spin-coated at 4000 rpm for 30 s under ambient conditions and preheated at 80 °C for 10 min. After cooling, a SnO_2_ solution doped with 50 mM NH_4_Cl was spin-coated 4000 rpm, 30 s and annealed at 150 °C for 30 min. The perovskite precursor solution Cs_0.05_(FA_0.85_MA_0.15_)_0.95_Pb­(I_0.85_Br_0.15_)_3_ and Spiro-OMeTAD solution were prepared according to our previous reports.^32,33^ The SnO_2_ films were treated with UV–ozone for 15 min before perovskite deposition. The perovskite film was deposited by spin coating at 4000 rpm for 25 s, with 375 μL of chlorobenzene dropped as an antisolvent at 18 s, followed by annealing at 100 °C for 60 min in a N_2_-filled glovebox. After cooling, CuM dispersed in 2-butanol at various concentrations (0, 0.025, 0.05, 0.075, and 0.1 mg/mL as shown in Figure S2) was spin-coated 4500 rpm, 20 s onto the perovskite surface and annealed at 100 °C for 10 min. Spiro-OMeTAD was then deposited 4500 rpm, 20 s and a 100 nm Au electrode was deposited by thermal evaporation.

#### 2.2.4 Characterization

The morphology of perovskite films and cross-sectional images of devices were examined using a Hitachi SU8230 scanning electron microscope (SEM). X-ray diffraction (XRD) patterns were collected on a Bruker D8 Advance diffractometer with Cu Kα radiation (λ = 1.5418 Å). X-ray photoelectron spectroscopy (XPS) measurements were performed using a Shimadzu/KRATOS AXIS SUPRA system. UV–vis absorption spectra were acquired with an Avantes fiber optic spectrometer equipped with a xenon light source. Surface topography was evaluated by Bruker JPK atomic force microscopy (AFM). Steady-state photoluminescence (PL) spectra were recorded with a DeltaFlex spectrometer (λ_exc_ = 405 nm, 10 kHz pulse). External quantum efficiency (EQE) and integrated *J*_sc_ were measured using a QE-R Enlitech system with a 75 W xenon lamp. J–V characteristics were measured with a Keithley 2450 SourceMeter under simulated AM1.5G illumination (100 mW/cm^2^, G2 V Pico LED solar simulator) with an active area of 0.09 cm^2^. Intensity-modulated photocurrent spectroscopy (IMPS) and electrochemical impedance spectroscopy (EIS) were conducted using a PAIOS system for electrical and optical characterization of solar cells and LEDs.

## 3 Results and Discussion

As illustrated in Figure 1 the device architecture consisted of glass/ITO/SnO_2_/perovskite/Spiro-OMeTAD/Au for the reference PSCs, while the modified devices incorporated an additional Cu_2_O–MIL-53­(Al) (CuM) interfacial layer deposited between the perovskite absorber and the HTL. In this study, CuM dispersed in 2-butanol was introduced onto the perovskite surface via spin coating to serve as a surface modification layer. The CuM treatment was designed to reduce lead migration from the perovskite, improve interfacial contact with the HTL, enhance charge extraction, and minimize nonradiative recombination losses at the perovskite/HTL interface.^16,19–21^

**Figure 1:**
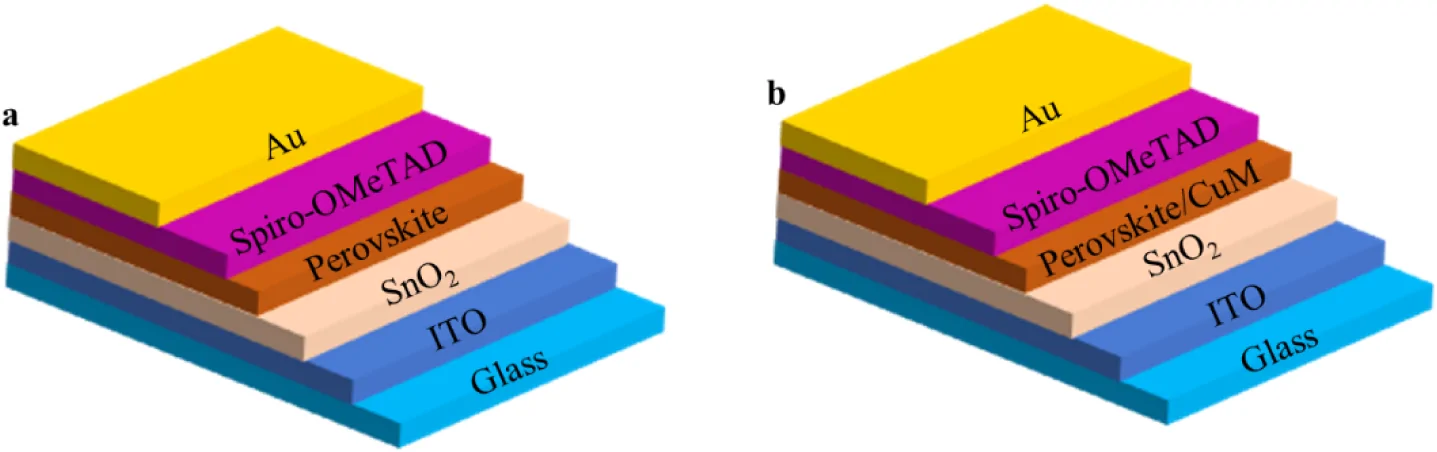
Schematic illustration of (a) a conventional perovskite solar cell (PSC) and (b) a PSC incorporating CuM surface treatment.

To evaluate the influence of CuM concentration on film quality, perovskite layers were treated with varying concentrations of CuM (0, 0.025, 0.05, 0.075, and 0.1 mg/mL) and characterized by top-view SEM imaging as shown in Figure 2. The pristine film as shown in Figure 2a displayed relatively irregular grain boundaries, while the films treated with 0.025, 0.05, 0.075, and 0.1 mg/mL CuM as shown in Figure 2b, 2c, 2d and 2e displayed visible white spot features, which are consistent with CuM-induced modification of the perovskite surface. This improved grain structure is expected to facilitate more efficient charge transport and reduced trap-assisted recombination, ultimately contributing to enhanced device performance in terms of open-circuit voltage, power conversion efficiency, and long-term stability.

**Figure 2:**
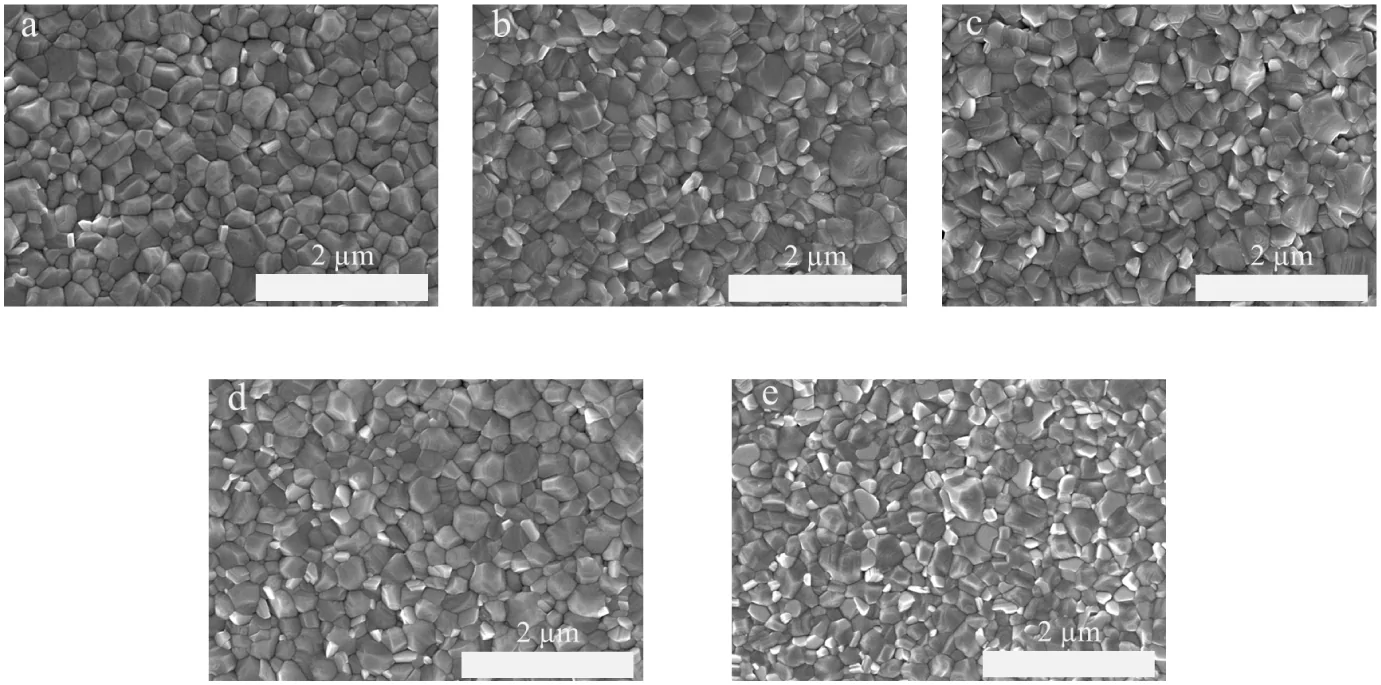
Top-view SEM images of perovskite films treated with different concentrations of Cu_2_O–MIL-53­(Al) (CuM): (a) pristine (0 mg/mL), (b) 0.025 mg/mL, (c) 0.05 mg/mL, (d) 0.075 mg/mL, and (e) 0.1 mg/mL.

To further examine the surface morphology associated with the white spots observed at the grain boundaries, the perovskite films were analyzed by atomic force microscopy (AFM), as shown in Figure 3. The AFM analysis revealed that the pristine film exhibited an average RMS roughness of 21.98 ± 1.35 nm as shown in Figure 3a, whereas, the 0.075 mg/mL CuM-treated film showed a slightly lower average RMS roughness of 18.86 ± 0.62 nm as shown in Figure 3b. Notably, the 0.075 mg/mL CuM-treated film also exhibited the lowest average RMS roughness among all investigated concentrations, as summarized in Figure S3. These results indicate that CuM treatment does not deteriorate the film surface and instead preserves a comparably smooth, and slightly more uniform, topography. The localized bright regions observed in the AFM images correspond to the white spots seen in SEM. To further clarify their origin, plan-view EDS analysis was performed for the pristine and CuM-treated films, as shown in Figure S4. After CuM treatment, distinct Cu and Al signals were detected, confirming the presence of Cu_2_O–MIL-53­(Al)-derived species on the film surface, while the Pb signal was attenuated relative to the pristine film. These results suggest that the bright surface features are more plausibly associated with CuM-containing surface domains and/or CuM-modified perovskite interfacial regions, rather than with residual PbI_2_-rich crystallites alone. This interpretation is consistent with the XRD results, where enhanced diffraction intensities at 14.2° (110) and 28° (220) were observed as shown in Figure 4a confirming the CuM-treated films exhibited increased preferred diffraction intensity.

**Figure 3:**
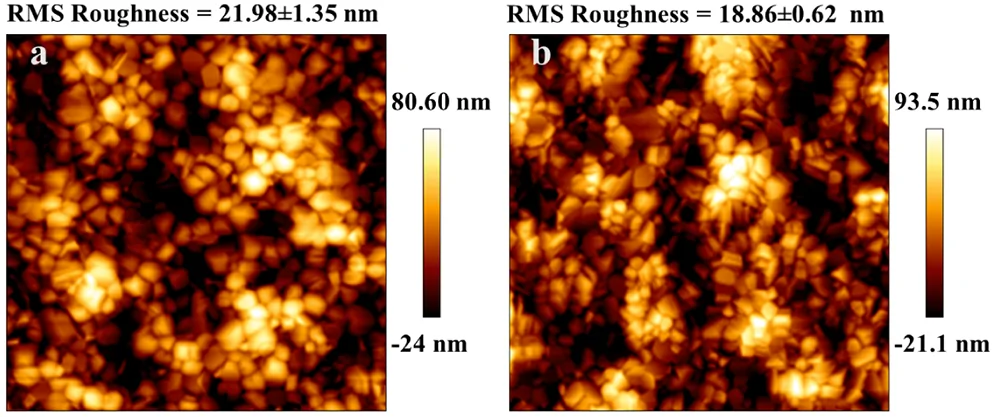
AFM topography images size 5 × 5 μm^2^ of perovskite films treated with (a) 0 mg/mL (pristine) and (b) 0.075 mg/mL CuM.

**Figure 4:**
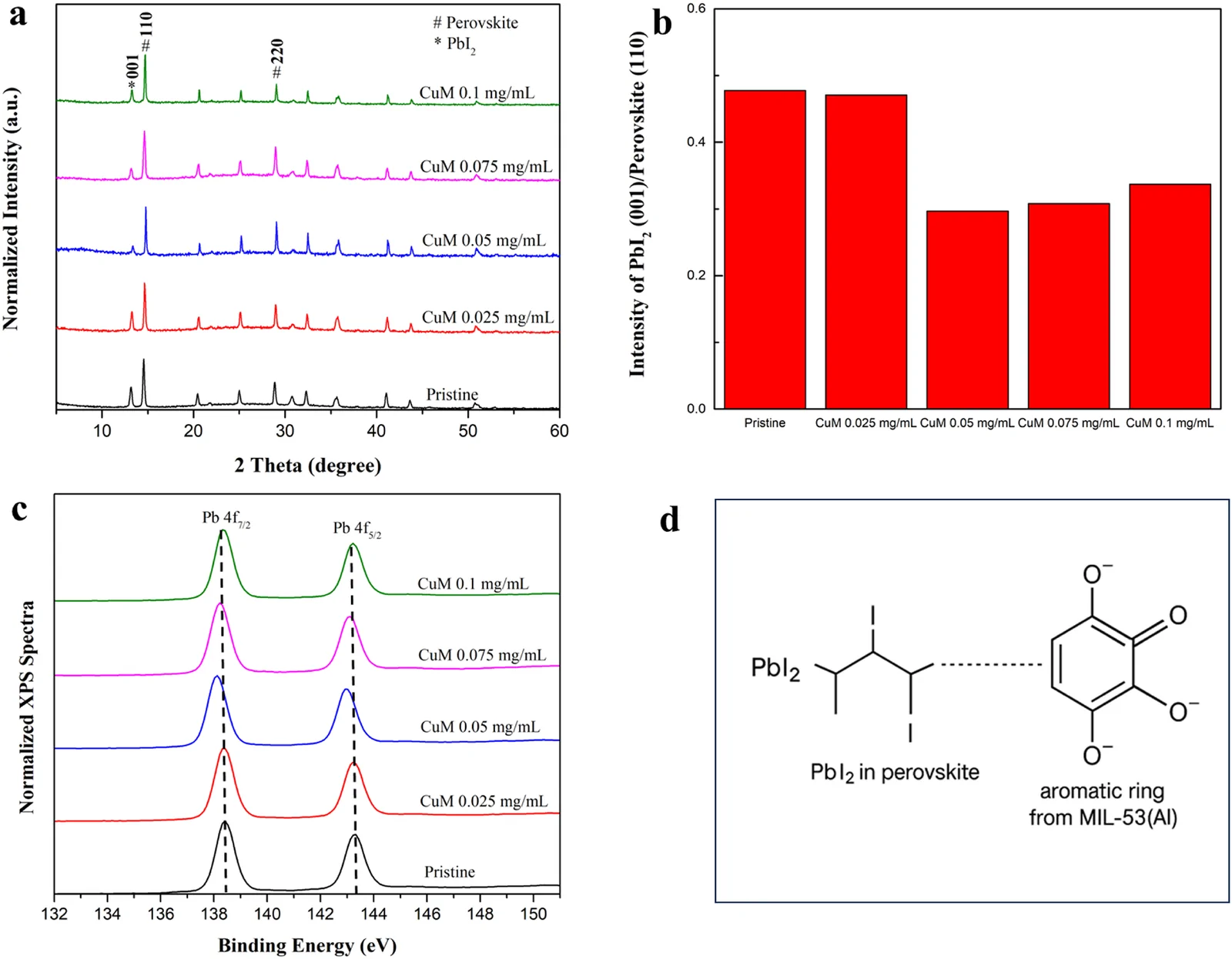
(a) XRD patterns, (b) quantitative comparison of the PbI_2_/perovskite diffraction intensity ratio, (c) Pb 4f peaks in XPS spectra of perovskite films with and without the CuM surface passivation and (d) chemical reaction between MIL-53­(Al) and PbI_2_.

Moreover, the XRD patterns of all perovskite films exhibited the characteristic diffraction peaks of the perovskite phase together with the PbI_2_ (001) reflection at 12.8°, as shown in Figure 4a. To enable a more quantitative comparison, the relative contribution of PbI_2_ was calculated from the maximum diffraction intensities of the PbI_2_ (001) and perovskite (110) as shown in Figure 4b. This analysis showed that the ratio decreased after CuM treatment compared with the pristine film, indicating a reduced relative PbI_2_-related diffraction contribution in the treated samples. The lowest ratio was observed for the 0.05 mg/mL CuM film, while the 0.075 mg/mL sample also showed a clearly reduced ratio relative to the pristine control. These results suggest that CuM treatment, particularly in the 0.05–0.075 mg/mL range, is effective in regulating PbI_2_-related surface residues, although the optimum photovoltaic performance is obtained at 0.075 mg/mL, reflecting the combined effects of interfacial passivation, carrier dynamics, and film quality rather than the PbI_2_/perovskite ratio alone. This observation is consistent with the XPS spectra as shown in Figure 4c, where the Pb 4f binding energies of CuM-modified films shifted toward lower values, indicating an increase in electron density around Pb^2+^, which is consistent with interfacial electronic interaction between the perovskite surface and the CuM layer. Such behavior may arise from interaction of the aromatic terephthalate ligands in MIL-53­(Al) with PbI_2_-related surface species as shown in Figure 4d, thereby modifying the local chemical environment and stabilizing the perovskite interface. These structural and electronic modifications are expected to reduce nonradiative recombination and facilitate more efficient charge transport at the perovskite/HTL interface, ultimately contributing to enhanced open-circuit voltage, power conversion efficiency, and device stability.

Moreover, the C 1s and O 1s spectra were also analyzed. In the C 1s region, the C–C component remains essentially unchanged at 285.01 eV, whereas the oxygen-containing components shift slightly toward higher binding energy after CuM treatment, with the C–O peak shifting from 286.62 to 286.86 eV and the C=O peak shifting from 288.57 to 288.65 eV as shown in Figure 5a. Similarly, in the O 1s region, the pristine film shows two oxygen-related components at 532.53 and 533.67 eV. which are assigned to C=O/carboxylate-related oxygen and C–O-related oxygen species, respectively. After CuM treatment these components slightly shift to higher binding energies of 532.64 and 533.75 eV as shown in Figure 5b indicating that the local chemical environment of oxygen-containing functionalities is perturbed after deposition of CuM on the perovskite surface. The fitted O 1s components exhibit fwhm values of 1.30–1.45 eV suggesting reasonable peak widths for the deconvolution. In addition, the overall R^2^ values are 0.941 and 0.897 for the pristine and CuM-treated films respectively as summarized in Table S1 confirming acceptable fitting quality. The slightly lower R^2^ value of the CuM-treated sample is attributed to the weak O 1s signal and relatively low signal-to-noise ratio arising from the ultrathin CuM modification layer. Taken together, the lower-binding-energy shift of Pb 4f, along with the slight positive shifts in the oxygen-containing C 1s and O 1s components, supports an interfacial electronic interaction involving the oxygen-containing terephthalate framework of MIL-53­(Al).

**Figure 5:**
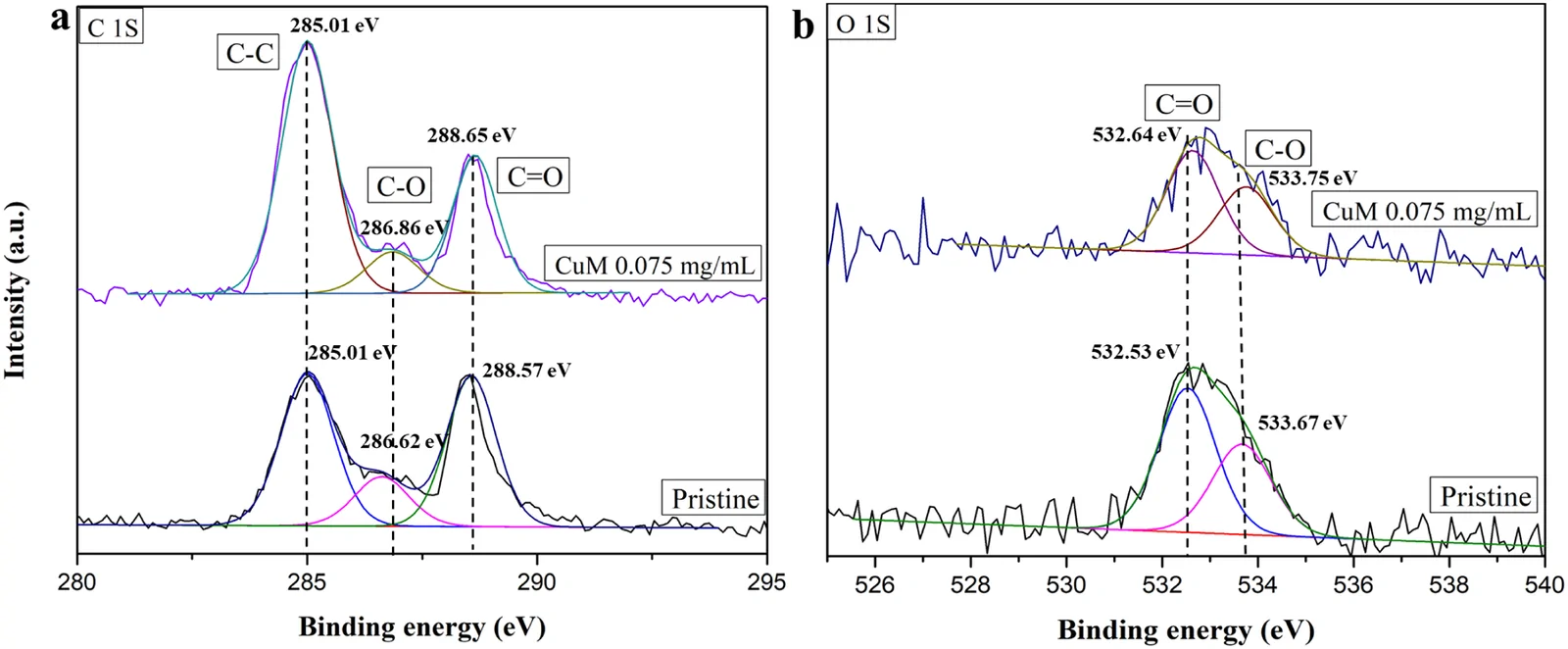
XPS spectra (a) C 1S and (b) O 1S of perovskite films with and without the CuM surface passivation.

To further probe the interfacial interaction between CuM and the perovskite surface, FTIR spectra of the pristine and 0.075 mg/mL CuM-treated films were compared. The CuM-treated film exhibited noticeable spectral changes relative to the pristine film in the regions around 600 cm^–1^, 1350–1470 cm^–1^, and 3270–3400 cm^–1^ as shown in Figure 6. The low-wavenumber change is consistent with modification of the local Pb–I lattice environment, while the midwavenumber region likely contains overlapping contributions from the FA/MA organic cations and the terephthalate framework of MIL-53­(Al). The broad high-wavenumber differences further indicate a modified N–H/O–H hydrogen-bonding environment after CuM treatment. In addition, comparison of the FTIR spectra of PbI_2_ and PbI_2_/CuM as shown in Figure S5 CuM introduces additional spectral contributions and alters the local chemical environment after contact with PbI_2_. These FTIR results are therefore presented as supportive evidence of interfacial chemical-environment modification, rather than as definitive proof of a specific chemical-bonding configuration.

**Figure 6:**
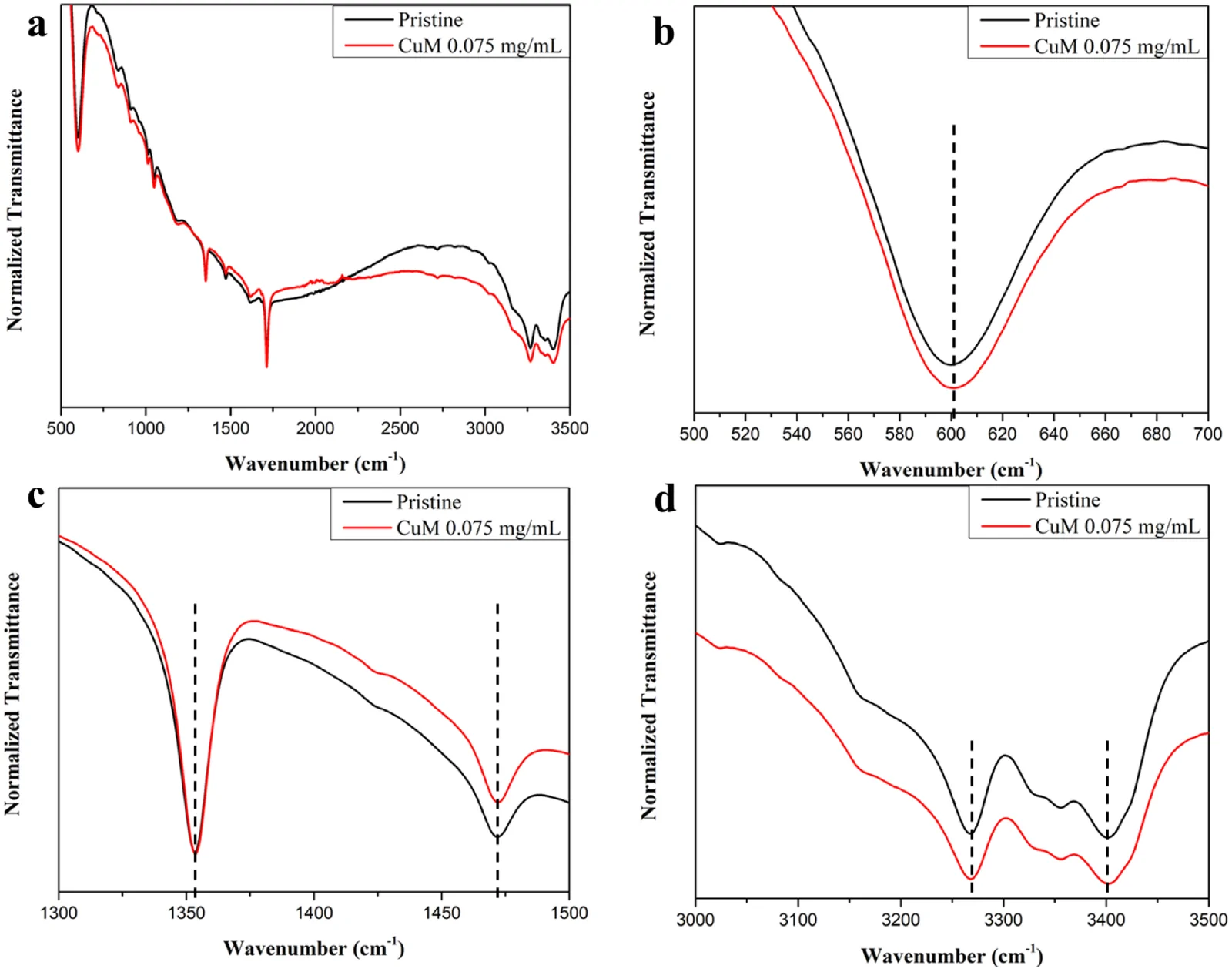
FTIR spectra of pristine and 0.075 mg/mL CuM-treated perovskite films: (a) full spectrum, (b) low-wavenumber region, (c) midwavenumber region and (d) high-wavenumber region.

The perovskite solar cells were fabricated in the conventional n–i–p configuration (ITO/SnO_2_/SnO_2_–NH_4_Cl/perovskite/CuM/Spiro-OMeTAD/Au) to evaluate the effect of CuM surface treatment at concentrations of 0, 0.025, 0.05, 0.075, and 0.1 mg/mL. The photovoltaic performance was assessed by current density–voltage (J–V) measurements under AM1.5G illumination as shown in Figure 7a. All devices exhibited negligible hysteresis between forward and reverse scans as summarized in Table S2. The best performance was obtained with 0.075 mg/mL CuM treatment, achieving a PCE of 20.25% with *V*_oc_ of 1.11 V, *J*_sc_ of 23.89 mA/cm^2^, and a fill factor (FF) of 76.51%. By comparison, the pristine device yielded a PCE of 18.68% (*V*_oc_ = 1.08 V, *J*_sc_ = 22.91 mA/cm^2^, FF = 75.53%), as summarized in Table 1. Statistical distributions across 16 devices are shown in Figure 7c further confirmed that the CuM-treated devices at 0.075 mg/mL consistently delivered higher efficiencies than both pristine and other CuM concentrations. In particular, both *V*_oc_ and FF showed notable improvements at this concentration, as illustrated in Figure S6. To further assess the role of the interfacial modifier, control devices treated with MIL-53­(Al) alone were compared with pristine and CuM-treated devices, and the corresponding J–V characteristics are shown in Figure S7. MIL-53­(Al) alone improved the *V*_oc_ and fill factor relative to the pristine device, indicating that the MOF component contributes to interfacial passivation. Upon incorporation of Cu_2_O into the MIL-53­(Al) framework, the CuM-treated device showed additional improvement, particularly in *J*_sc_, leading to the best overall photovoltaic performance among the tested conditions. These results indicate that Cu_2_O incorporation provides further benefit beyond MIL-53­(Al) treatment alone under the present processing conditions. To place the present device performance in the proper literature context, a benchmarking comparison with reported MOFs interfacial modification strategies is provided in Table S3.

**Figure 7:**
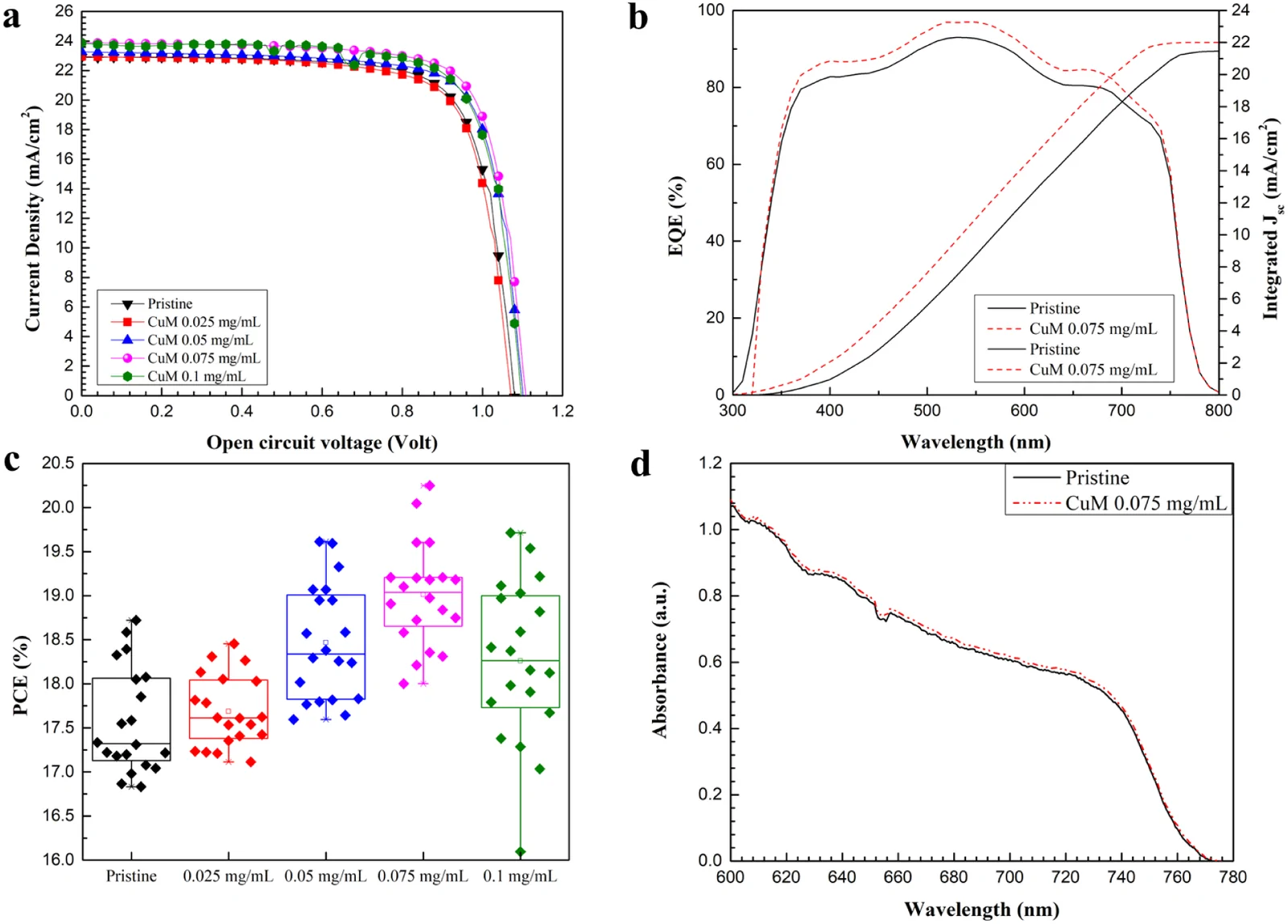
(a) J–V characteristics and (b) EQE spectra of the best PSCs with pristine and concentration of CuM at 0.075 mg/mL on surface treatment on perovskite solar cells (c) summarizes the distribution of the devices PCE of CuM at concentration 0, 0.025, 0.05, 0.075, and 0.1 mg/mL CuM surface treatment of perovskite solar cells from16 devices. (d) UV–vis absorption spectra of pristine and 0.075 mg/mL CuM surface treatment perovskite films.

**Table 1:** Reverse Scan Photovoltaic Parameters for the PSCs with Different Concentration of CuM at 0, 0.025, 0.05, 0.075, and 0.1 mg/mL on Surface Treatment on Perovskite Solar Cells

Concentration(mg/mL)	*V*_oc_ (V)	*J*_sc_ (mA/cm^2^)	FF	PCE (%)
Pristine	1.08 (1.06 ± 0.02)	22.91 (23.47 ± 0.30)	75.53 (72.00 ± 1.23)	18.68 (17.73 ± 0.56)
CuM 0.025	1.07 (1.06 ± 0.01)	22.94 (23.04 ± 0.14)	75.12 (73.74±0.91)	18.46 (17.81 ± 0.35)
CuM 0.05	1.10 (1.08 ± 0.02)	23.27 (23.69 ± 0.75)	76.53 (74.09±1.53)	19.62 (18.66 ± 0.58)
CuM 0.075	1.11 (1.09 ± 0.02)	23.89 (23.45 ± 0.37)	76.51 (75.51±0.81)	20.25 (19.21 ± 0.46)
CuM 0.1	1.10 (1.08 ± 0.02)	23.88 (23.44 ± 0.19)	75.25 (73.69 ± 1.59)	19.71 (18.59 ± 0.63)

The external quantum efficiency (EQE) spectra and integrated *J*_sc_ values are shown in Figure 7b. Devices incorporating 0.075 mg/mL CuM exhibited stronger EQE intensity across the entire spectral range relative to pristine PSCs, consistent with the enhanced *J*_sc_ values. Furthermore, UV–vis absorption spectra revealed that the CuM-modified perovskite films displayed higher absorbance intensity without any shift in the absorption edge as shown in Figure 7d, indicating that CuM surface treatment improves light harvesting without altering the perovskite bandgap similar other material coated on perovskite layer has been reported by Yang, Yuxuan et al.^34^ The simultaneous improvement in EQE response and absorption intensity can be attributed to more efficient charge extraction and reduced interfacial recombination, in agreement with the structural and interfacial modifications observed in XRD, XPS, and SEM analyses. Collectively, these results demonstrate that CuM functions as an effective interfacial passivation layer, suppressing defect-mediated recombination and facilitating efficient carrier transport, which directly translates into higher open-circuit voltage, fill factor, and overall power conversion efficiency, as well as improved device stability. To gain deeper insight into the underlying charge-transfer processes responsible for these enhancements, steady-state and time-resolved photoluminescence (PL/TRPL) measurements were carried out as shown in Figure 8.

**Figure 8:**
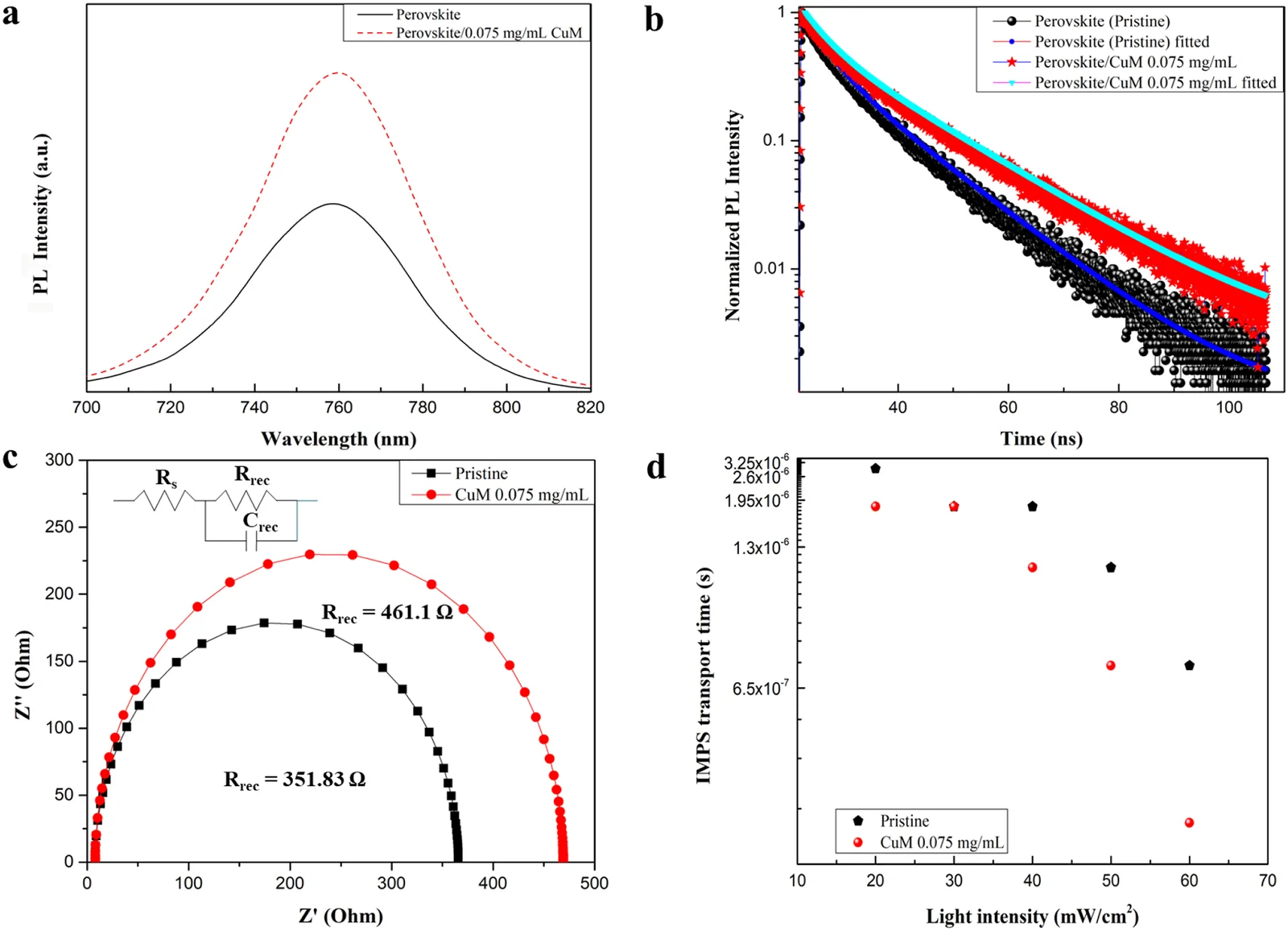
(a) Steady-state PL spectra and (b) time-resolved photoluminescence (TRPL) decay of ITO/perovskite pristine and CuM 0.075 mg/mL surface treatment (c) R_rec_ calculated from the Nyquist plot and (d) intensity-modulated photocurrent spectroscopy (IMPS) transport time constant of the with pristine perovskite and 0.075 mg/mL CuM devices.

The charge-transfer dynamics at the perovskite/CuM interface were examined using steady-state and time-resolved photoluminescence (PL and TRPL) measurements under 405 nm excitation as shown in Figure 8a and 8b. Relative to the pristine perovskite film, the CuM-modified film (0.075 mg/mL) exhibited markedly higher PL intensity, indicative of effective passivation of nonradiative recombination centers. TRPL analysis further supported this result, showing that the average carrier lifetime (τ_ave_) increased from 8.30 ns in the pristine film to 11.45 ns in the CuM-treated film as summarized in Table S4, confirming suppressed trap-assisted recombination and enhanced charge carrier generation. In addition, when coupled with Spiro-OMeTAD, the CuM-modified perovskite film showed reduced PL quenching relative to the pristine perovskite film, the CuM-modified film (0.075 mg/mL) exhibited higher PL intensity, indicating effective suppression of nonradiative recombination. TRPL analysis further supported this result, with the average carrier lifetime increasing from 8.30 ns for the pristine film to 11.45 ns for the CuM-treated film. In addition, for the HTL-containing stacks, the ITO/perovskite/CuM/Spiro-OMeTAD sample exhibited stronger PL quenching and a markedly shorter average lifetime (0.15 ns) than the ITO/perovskite/Spiro-OMeTAD control (0.89 ns), consistent with faster interfacial hole transfer after CuM treatment as shown in Figure S8.Together, These results support the dual role of CuM in defect passivation and improved interfacial charge extraction. To further examine the device structure after CuM incorporation, cross-sectional SEM images were collected for both pristine and CuM-modified PSCs, as shown in Figure S9. All functional layers, including glass/ITO, SnO_2_, perovskite, Spiro-OMeTAD, and Au, are clearly distinguishable, confirming the complete device architecture. Importantly, the incorporation of CuM at 0.075 mg mL^–1^ does not noticeably alter the thickness of the perovskite absorber layer. Instead, the CuM-modified device maintains a continuous and well-defined perovskite/HTL interface, suggesting that the ultrathin CuM treatment is compatible with subsequent Spiro-OMeTAD deposition. Together with the PL/TRPL results, this cross-sectional observation further supports improved interfacial electronic properties and suppressed charge recombination in the CuM-treated PSCs. These findings directly correlate with the improved *V*_oc_, FF, and PCE observed in CuM-treated devices as shown Figure 7 and Table 1, underscoring the crucial role of CuM in interfacial passivation and charge extraction.

Electrochemical impedance spectroscopy (EIS) was employed to investigate the charge-transport dynamics of pristine and CuM-modified 0.075 mg/mL PSCs. The Nyquist plots, displaying the real and imaginary components of impedance, are presented in Figure 8c the semicircle diameter of the CuM-treated device was noticeably larger than that of the pristine device, indicating improved resistance to recombination. The spectra were fitted using an equivalent circuit comprising three resistors-series resistance (R_s_) and recombination resistance (R_rec_) together with one capacitor representing recombination capacitance (C_rec_) as shown in Table S5. The CuM-treated device exhibited a higher R_rec_ value of 461.1 Ω compared to 351.83 Ω for the pristine device, indicating suppressed charge recombination and improved interfacial carrier dynamics at the perovskite/HTL interface.

To further probe charge transport and recombination, intensity-modulated photocurrent spectroscopy (IMPS) was performed. Measurements were conducted under varied light intensities from 20 to 60 mW/cm^2^ with a fixed offset voltage of 0.9 V. The frequency peak (f_peak_) in the Bode plot of the imaginary component as shown in Figure S10 was used to calculate the charge-transport time constant (τ_tr_) according to eq 1. As shown in Figure 8d, the IMPS transport times decreased with increasing light intensity, and the CuM-treated PSC consistently exhibited shorter τ_tr_ values compared to the pristine device. This result is consistent with improved carrier transport dynamics and reduced recombination losses in the CuM-treated devices, thereby contributing to the enhanced photovoltaic performance. These findings align with the PL/TRPL analyses and J–V characteristics, supporting that CuM surface modification effectively passivates trap states and suppresses recombination while improving interfacial carrier dynamics, leading to higher *V*_oc_, improved FF, and enhanced PCE.

1 $$\tau_{tr}=\frac{1}{2\pi f_{peak}}$$

Furthermore, the long-term stability of unencapsulated PSCs was evaluated according to the ISOS-D1 protocol. Devices were stored in the dark at 20–25 °C in a class 10,000 cleanroom under ambient relative humidity of 40–60%, and J–V measurements were performed once per week until the total aging time reached 1000 h. All CuM-treated devices exhibited improved normalized PCE retention relative to the pristine control, indicating that CuM treatment enhances ambient storage stability over the investigated concentration range. Among these devices, the PSC treated with 0.075 mg/mL CuM showed the highest overall retention throughout the aging period, maintaining 96.56% of its initial efficiency after 1000 h, whereas the pristine device retained only 78.66% under the same conditions. The devices treated with 0.025, 0.05, and 0.1 mg/mL CuM also outperformed the pristine control, but their long-term retention remained lower than that of the 0.075 mg/mL device as shown in Figure 9a. In addition, thermal-storage stability was examined by aging the pristine and 0.075 mg/mL CuM-treated devices in the dark at 65 °C under N_2_. Under these conditions, the CuM-treated device consistently exhibited 93% PCE retention but the pristine device retained only 77% throughout the test period as shown in Figure 9b, further confirming the beneficial role of CuM in improving device durability under thermal stress. Taken together, these results indicate that the optimum CuM concentration is determined not only by photovoltaic efficiency, but also by the balance between interfacial passivation and long-term stability. Overall, the enhanced retention is consistent with the role of CuM in mitigating PbI_2_-related interfacial degradation and suppressing nonradiative recombination, thereby improving both ambient and thermal storage stability within the scope of the present study.

**Figure 9:**
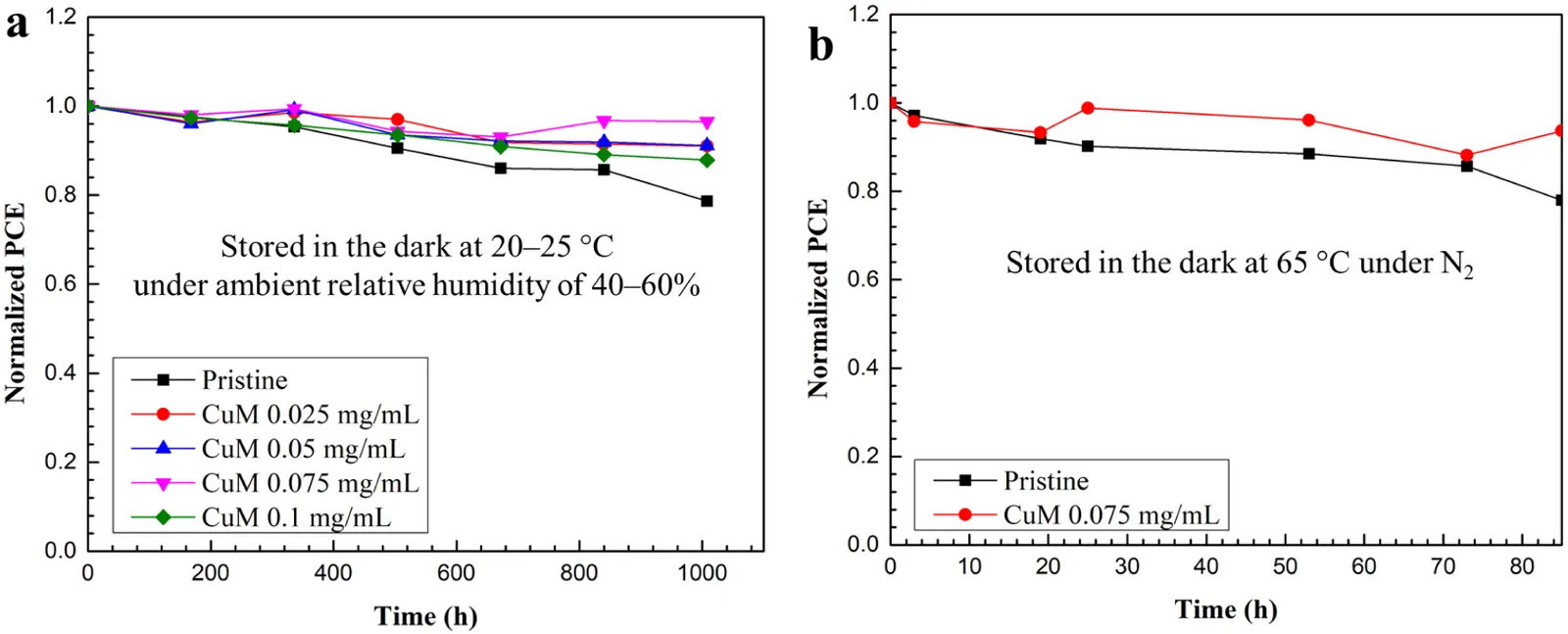
(a) Long-term stability of PSCs for 1000 h under humidity 40–60%RH unencapsulation. And (b) Thermal stability of PSCs at 65 °C under N_2_.

## 4 Conclusions

In summary, we have demonstrated that surface modification of perovskite films using a small amount of Cu_2_O–MIL-53­(Al) (CuM) provides an effective strategy to simultaneously enhance efficiency and stability in perovskite solar cells. Spin-coated CuM serves as regulate PbI_2_-related surface residues, perturbs the local interfacial chemical environment, and improves the perovskite/HTL junction. Optical measurements confirmed that CuM treatment does not alter the absorption edge of the perovskite film, thereby preserving the bandgap, while steady-state PL and TRPL revealed extended carrier lifetimes consistent with reduced trap-assisted recombination. EIS and IMPS analyses further corroborated these findings by showing higher recombination resistance and shorter transport times in CuM-modified devices. Thereby, contributing to enhanced photovoltaic performance and device durability. The optimized treatment condition 0.075 mg/mL delivered the best overall balance of efficiency 20.25% of PCE with negligible hysteresis while also showing improved ambient and thermal storage stability relative to the pristine control. Demonstrating the strong stabilize effect of CuM surface passivation. Collectively, these results the combined balance of interfacial chemical modification, defect passivation, carrier dynamics, and long-term stability. These insights provide a useful foundation for future development of CuM-type surface treatments toward more durable device architectures and larger-area perovskite modules.

## Supplementary Material



## Notes

TEM morphology, dispersion behavior of CuM, AFM topography, top-view SEM images and EDS pattern of perovskite, FTIR spectra of PbI2, photovoltaic parameters versus CuM concentration, J–V characteristics of the PSC devices, PL spectra, IMPS Bode plots, summarizes the O 1s XPS deconvolution parameters, photovoltaic performance parameters, application of MOFs in interfacial layer of PSCs, TRPL decay data and EIS extracted parameters (PDF)
