# Supplementary material for: Perovskite Surface Treatment with Cu2O‑MIL-53 (Al) Metal-Organic Frameworks for Enhanced Efficiency and Stable Perovskite Solar Cells
Source: ACS Omega. 2026 Aug 12;11(33):48972–82. doi: 10.1021/acsomega.5c12575 (PMC13520477; doi:10.1021/acsomega.5c12575)
Supplement: Supplementary file 1 [file ao5c12575_si_001.pdf]

Supporting Information

Perovskite Surface treatment with Cu_2O-MIL-53 (Al) Metal Organic Frameworks for Enhanced Efficiency and stable Perovskite Solar Cells

Sorrawit Meeklinhom[a], Madsakorn Towannang[b,], Jirasak Gamonchuang[a], Kitiphat Sinthiptharakoon[a], Komsun Lapawae[a], Nuttaya Sukgorn[a], Nopporn Rujisamphan[c], Chalida Imhan[a], Chalita Ratanatawanate[a], Pipat Ruankham[d], Duangmanee Wongratanaphisan[b,d], Pongsakorn Kanjanaboos[e], Anusit Kaewprajak[a], Pisist Kumnorkaew[a,*]*

AUTHOR ADDRESS

[a] National Nanotechnology Center (NANOTEC), National Science and Technology Development Agency (NSTDA), 111 Thailand Science Park, Pathum Thani, 12120, Thailand; sorrawit.mee@ncr.nstda.or.th(S.M.);Jirasak.gam@ncr.nstda.or.th(J.G.);nuttaya.suk@ncr.nstda.or.th (N.S.); anusit@nanotec.or.th (A.K.); pisist@nanotec.or.th (P.K.); chalida.imh@ncr.nstda.or.th (C.I.); chalita@nanotec.or.th. (C.R.) kitiphat@nanotec.or.th (K.S.); komsun.lapawae@gmail.com (K.L.)

[b] Thailand Center of Excellence in Physics (ThEP center), Ministry of Higher Education, Science, Research and Innovation, Bangkok, 10400, Thailand; madsakorn@gmail.com (M.T.); duangmanee.wong@cmu.ac.th (D.W.)

[c] Nanoscience and nanotechnology Graduate Program Faculty of Science King Mongkut's University of Technology Thonburi Bangkok 10140, Thailand; nopporn.ruj@kmutt.ac.th (N.R.)

[d] Department of Physics and Materials Science, Faculty of Science, Chiang Mai University, Chiang Mai 50200, Thailand; duangmanee.wong@cmu.ac.th (D.W.); pipat.r@cmu.ac.th (P.R)

[e] School of Materials Science and Innovation, Faculty of Science, Mahidol University, Nakhon Pathom 73170, Thailand; pongsakorn.kan@mahidol.edu (P.K.)

*Email:; madsakorn@gmail.com; pisist@nanotec.or.th

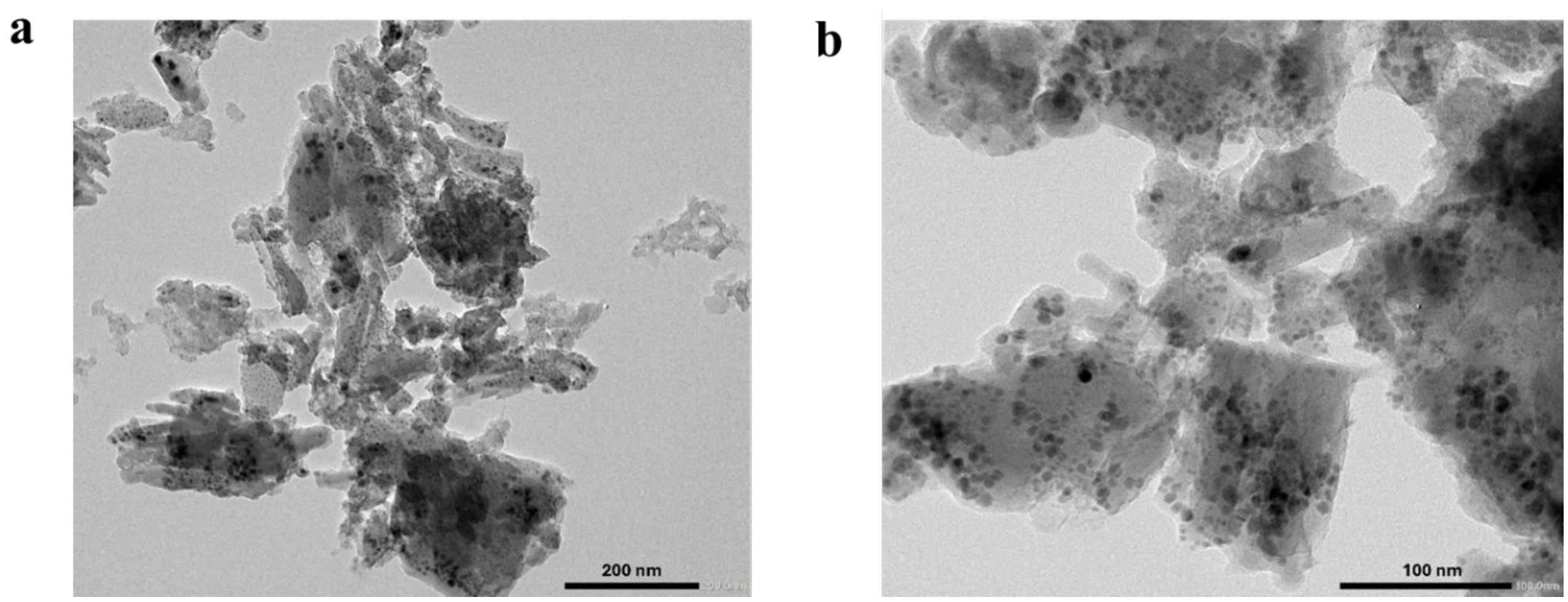

Figure S1. TEM images of (a) CuM composite (magnification of 50k), and (b) CuM composite (magnification of 120k).

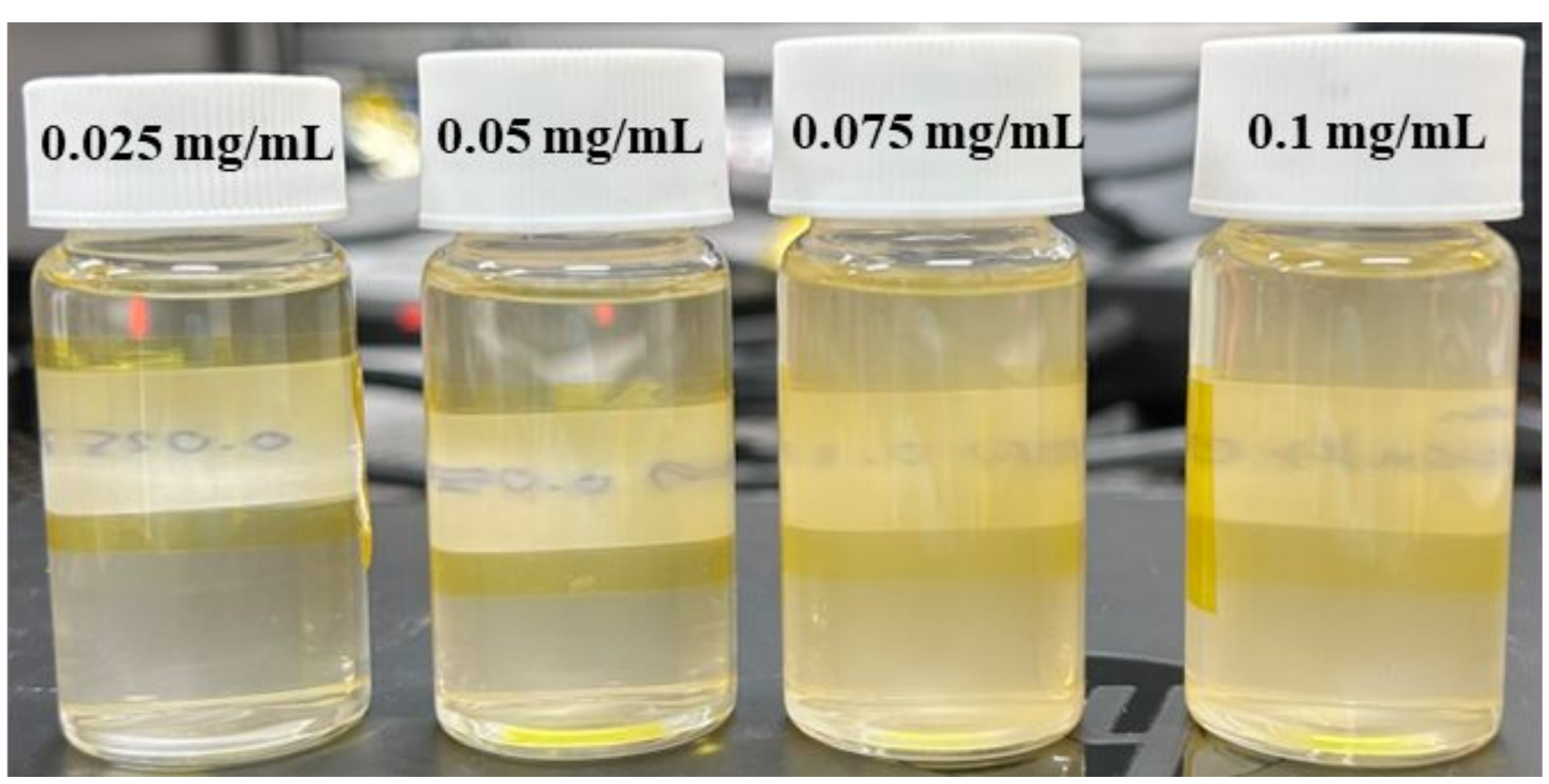

Figure S2. CuM dispersed in 2-butanol at various concentrations (0.025, 0.05, 0.075, and 0.1 mg/mL).

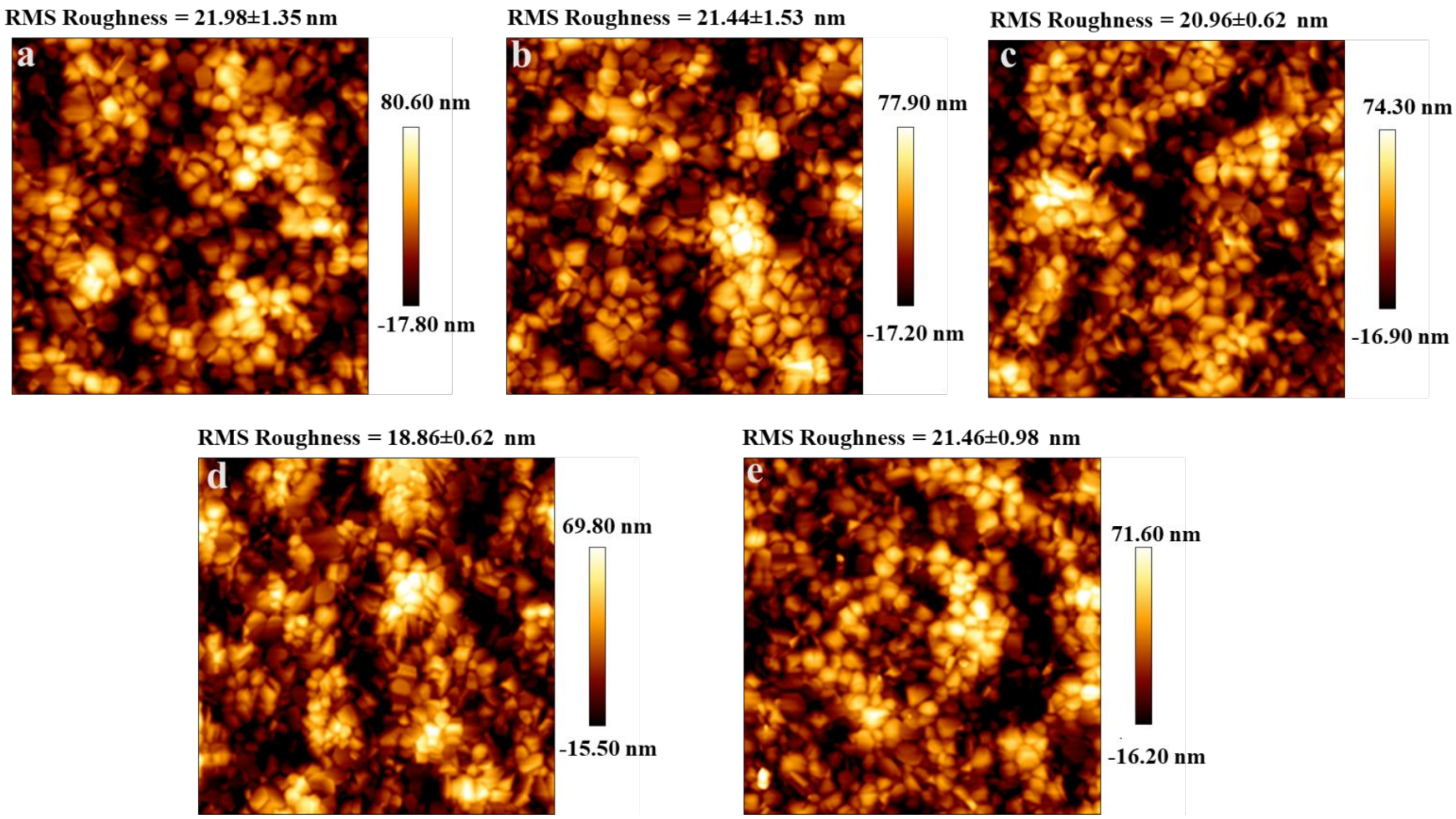

Figure S3. AFM topography images size 5x5 μm^2 of perovskite films treated with (a) pristine (0 mg/mL), (b) 0.025 mg/mL, (c) 0.05 mg/mL, (d) 0.075 mg/mL and (e) 0.1 mg/mL.

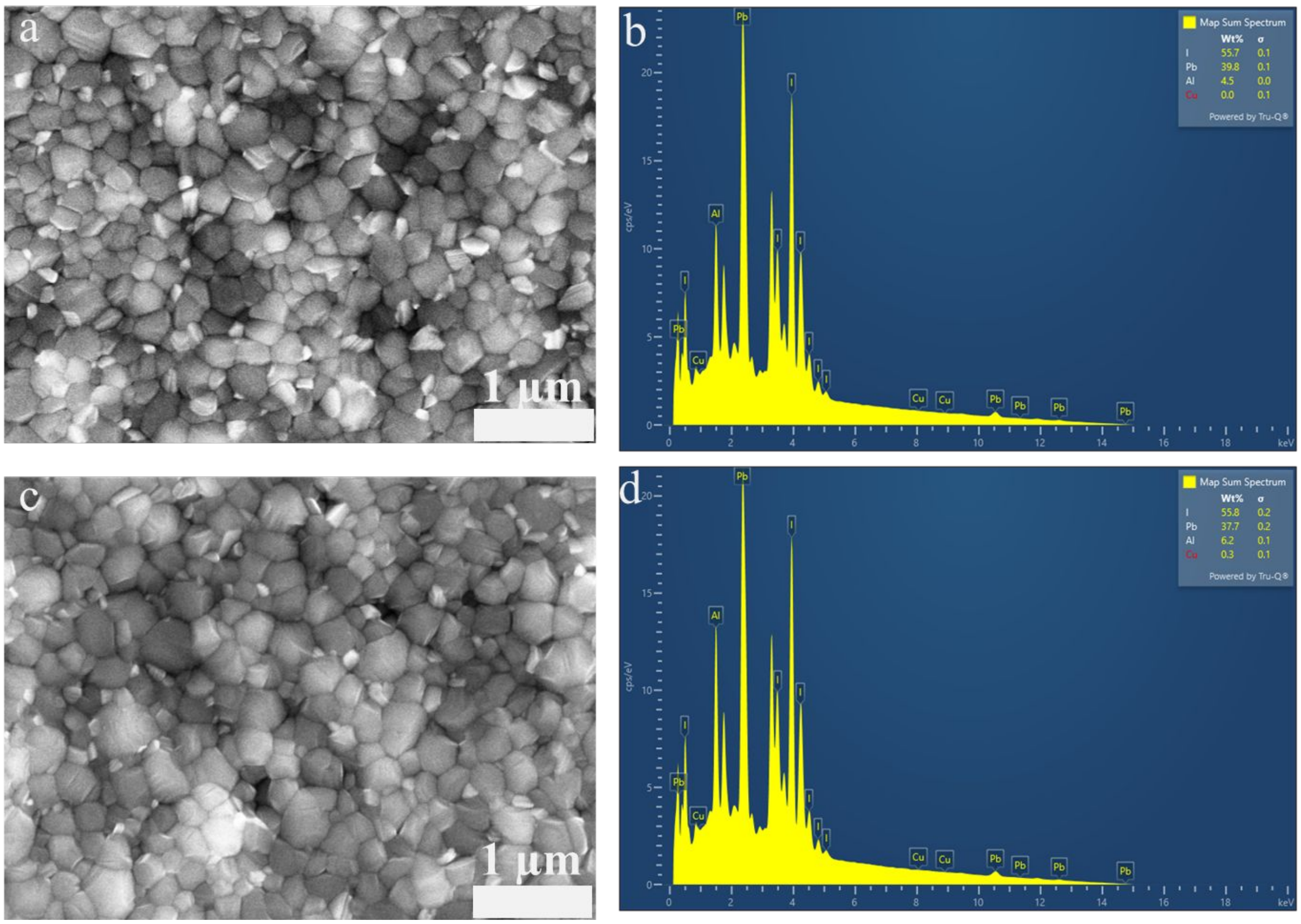

Figure S4. Top-view SEM images of perovskite films treated with different concentrations of CuM (a) pristine (0 mg/mL) and (c) 0.075 mg/mL. EDS pattern of perovskite films treated with different concentrations of CuM (b) pristine (0 mg/mL) and (d) 0.075 mg/mL.

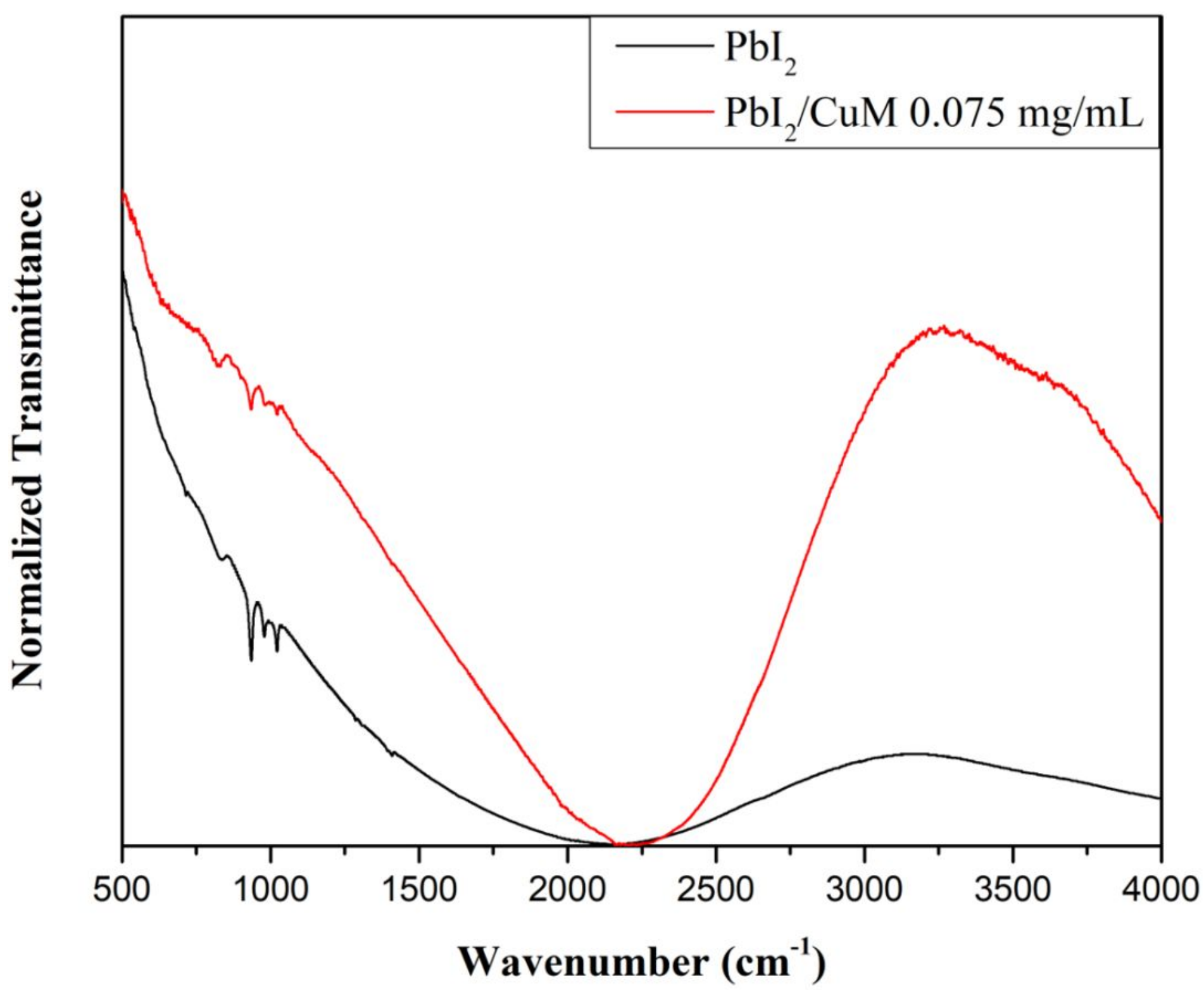

Figure S5. FTIR spectra of PbI_2 and PbI_2 0.075 mg/mL CuM-treated film.

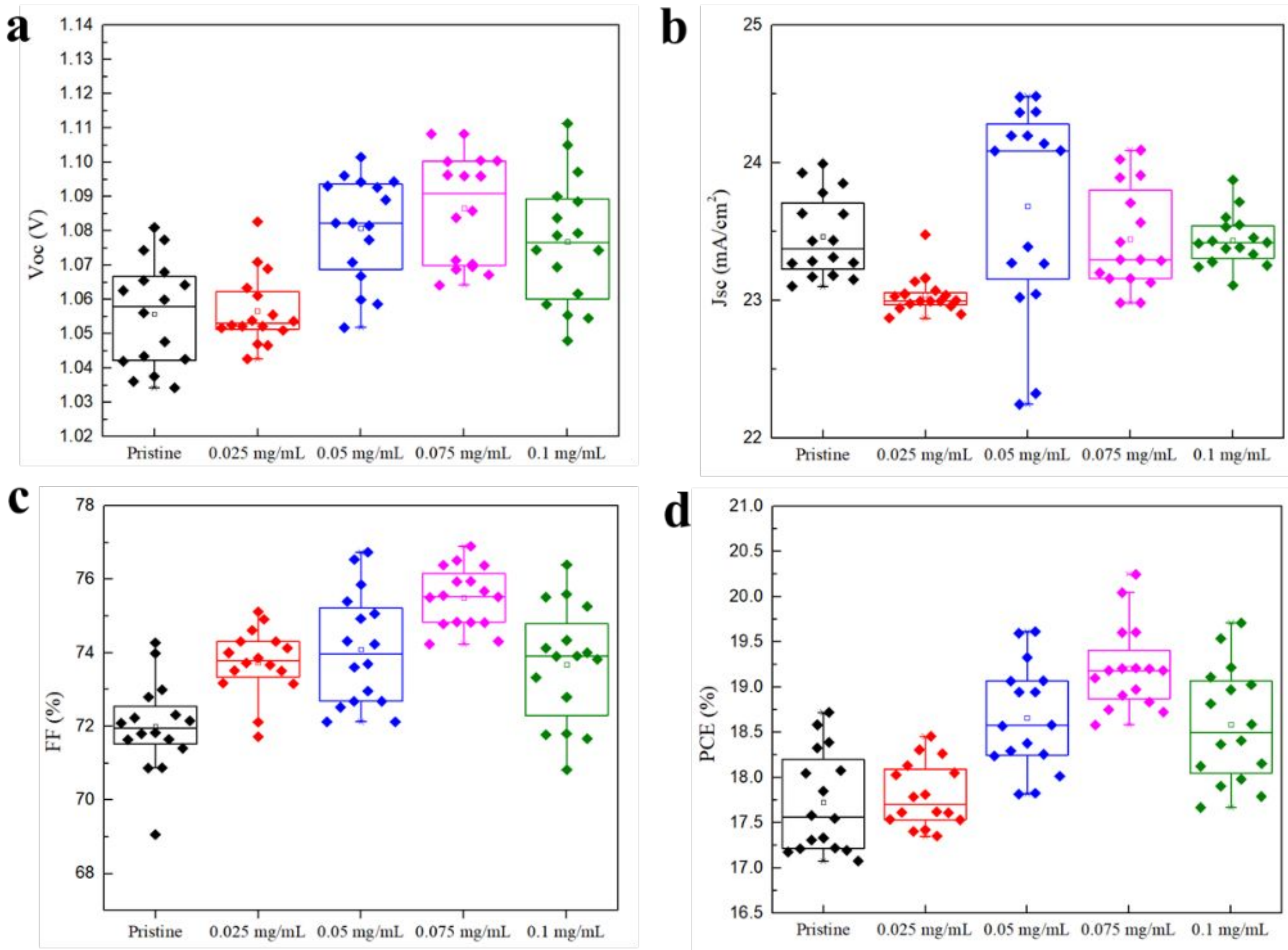

Figure S6. Summarizes the distribution of photovoltaic parameters of the devices CuM at concentration of CuM at 0, 0.025, 0.05, 0.075 and 0.1 mg/mL on perovskite layer of perovskite solar cells from 16 devices.

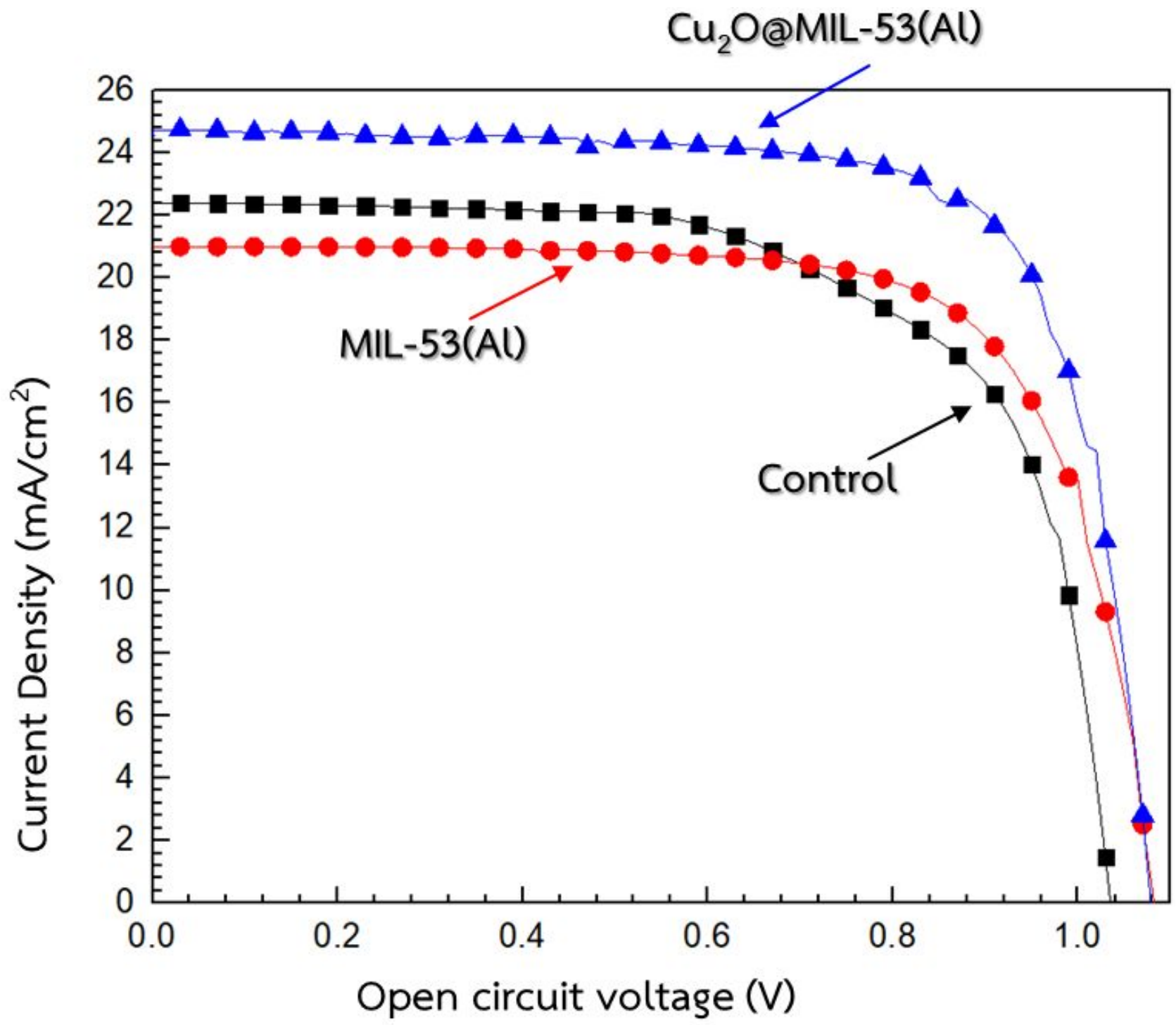

Figure S7. J−V characteristics of the PSCs devices with control (pristine), concentration of MIL-53(Al) 0.075 mg/mL and concentration of CuM at 0.075 mg/mL

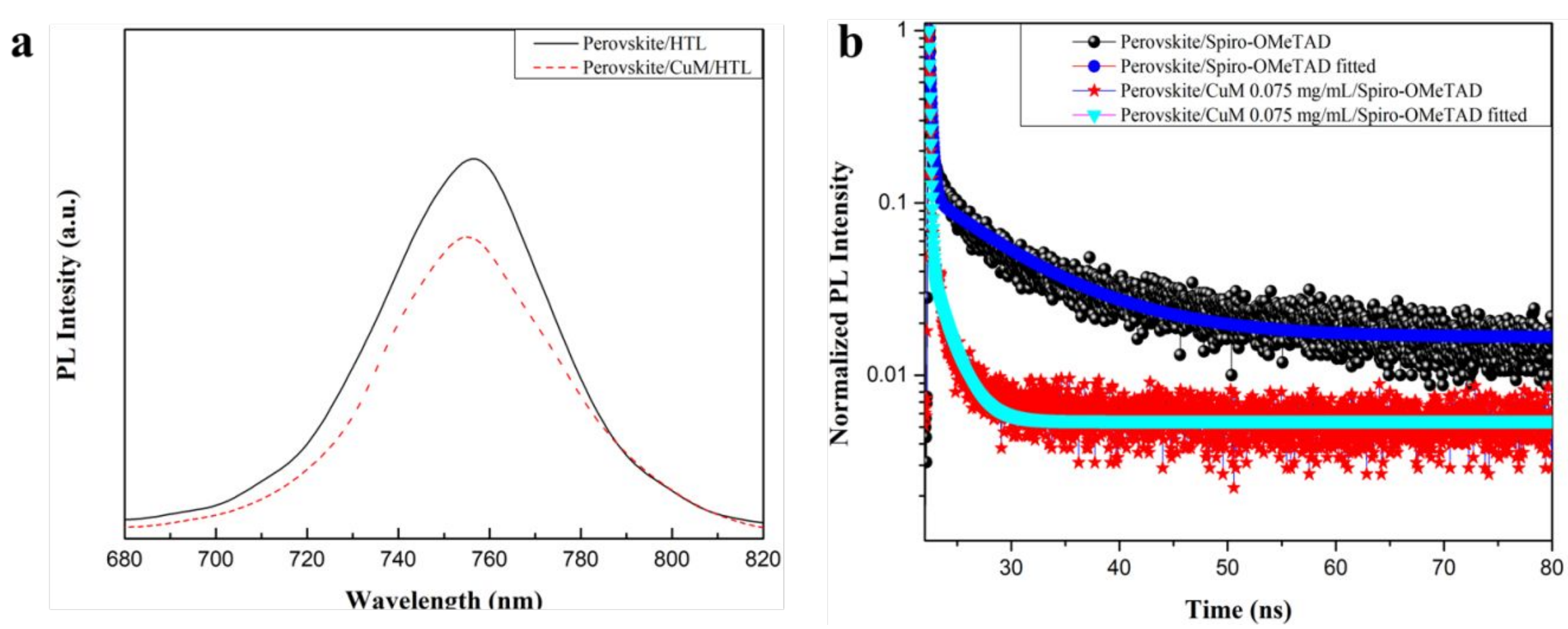

Figure S8. (a) Steady-state PL spectra of ITO/perovskite/Spiro-OMeTAD and ITO/perovskite/ 0.075 mg/mL CuM /Spiro-OMeTAD. (b) time-resolved photoluminescence (TRPL) decay of ITO/perovskite/Spiro-OMeTAD and ITO/perovskite/ 0.075 mg/mL CuM /Spiro-OMeTAD.

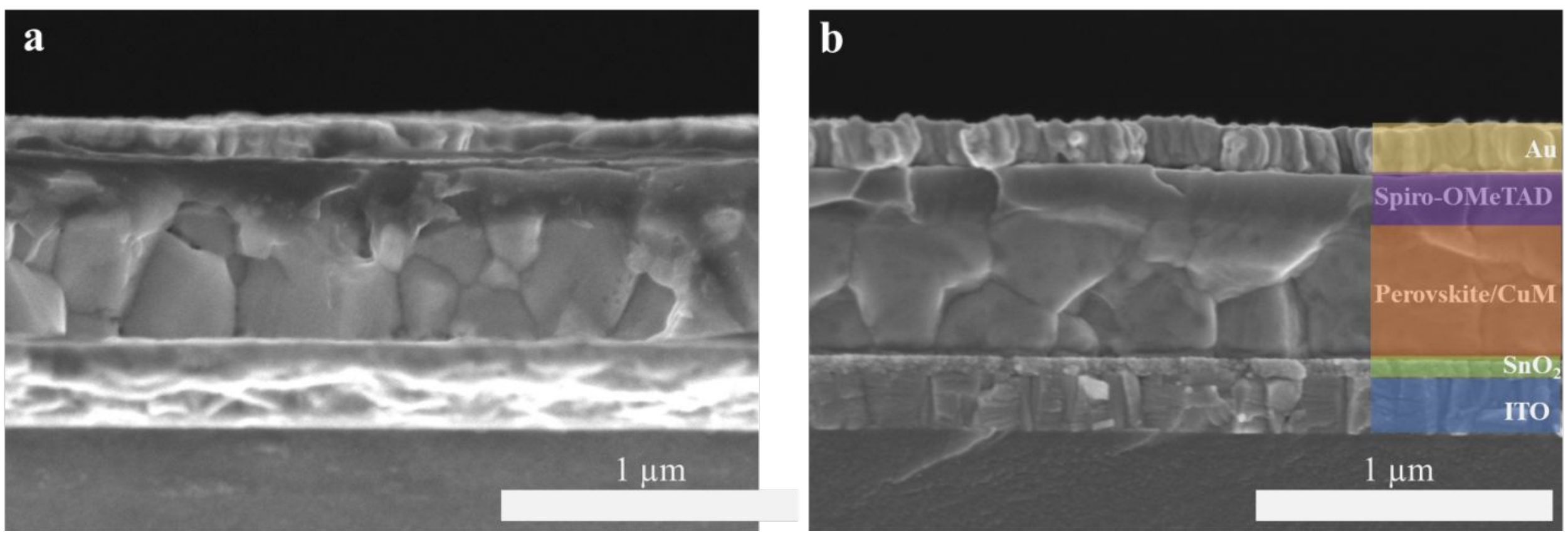

Figure S9. cross-sectional SEM image of perovskite solar cells (a) pristine and (b) 0.075 mg/mL of CuM surface treatment.

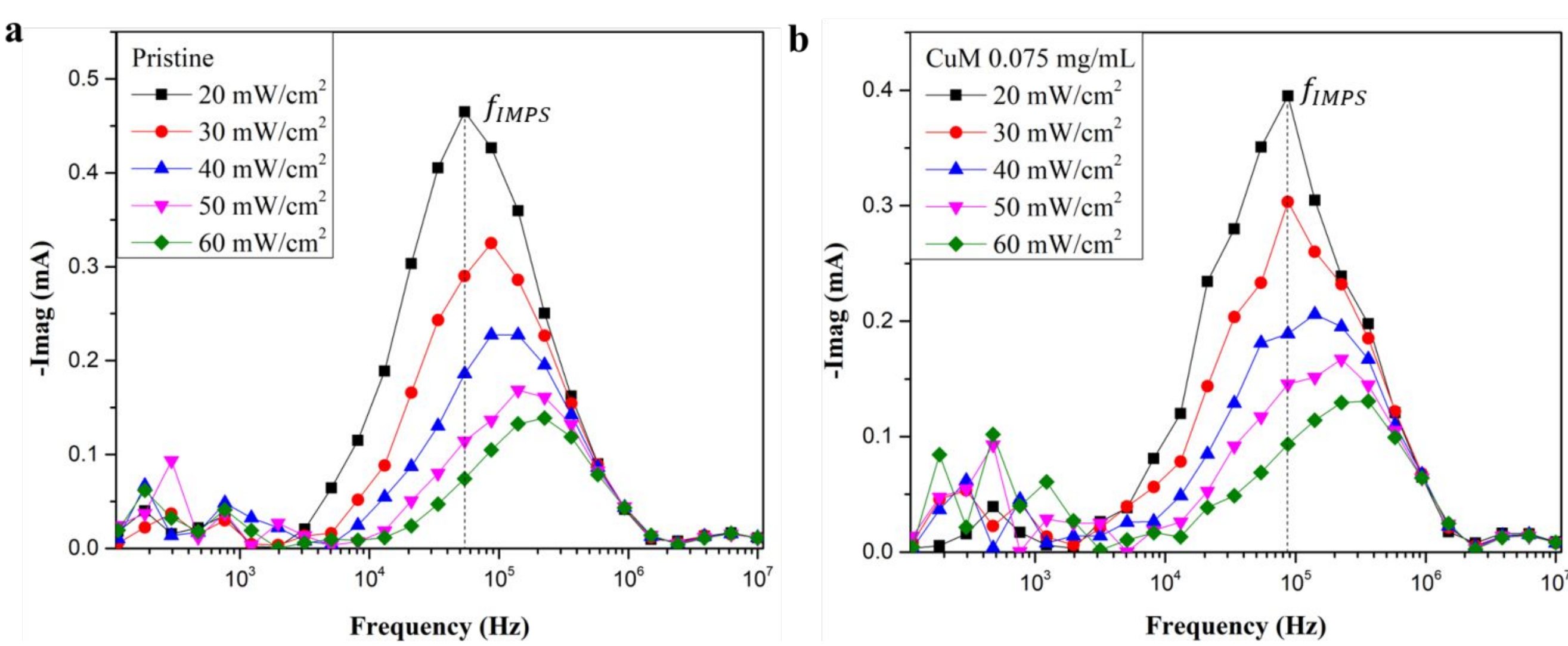

Figure S10. IMPS Bode type plots of imaginary current part of perovskite devices with (a) pristine device and (b) 0.075 mg/mL CuM surface treatment as a function of various illumination intensities (20, 30, 40, 50, and 60 mW/cm^2).

Table S1 Summarizes the O 1s XPS deconvolution parameters of pristine and CuM-treated perovskite films.

Sample	Binding energy (eV)	Assignment	FWHM	Area%	Overall R^2
Pristine	532.53	C=O	1.40	60.67	0.941
Pristine	533.67	C-O	1.45	39.33	0.941
CuM 0.075 mg/mL	532.64	C=O	1.30	59.13	0.897
CuM 0.075 mg/mL	533.75	C-O	1.35	40.87	0.897

Table S2. Summary of photovoltaic performance parameters for devices with different CuM concentrations.

Concentration(mg/mL)	Scan direction	V_{oc} (V)	J_{sc} (mA/cm^2)	*FF* (%)	*PCE* (%)
Pristine	Reverse	1.08	22.91	75.53	18.68
	Forward	1.07	22.76	75.03	18.32
	Reverse	1.08	23.03	73.72	18.27

CuM 0.025	Forward	1.07	22.94	75.12	18.46
CuM 0.05	Reverse	1.10	23.39	75.39	19.33
	Forward	1.10	23.27	76.53	19.62
CuM 0.075	Reverse	1.10	24.03	75.52	20.05
	Forward	1.11	23.89	76.51	20.25
CuM 0.1	Reverse	1.10	23.88	75.25	19.71
	Forward	1.11	23.72	71.80	18.82

Table S3. Application of Metal-organic frameworks (MOFs) in interfacial Layer of PSCs

MOFs	V_{oc} (V)	J_{sc} mA/cm^2	FF (%)	PCE (%)	Stability	Ref
MOF-808	1.06	21.01	79.8	17.81	80% Retained	[1]
UiO-66	1.07	21.85	76.9	18.01	70% Retained	[1]
Cr-MOF	1.16	23.84	83.0	23.06	91.2% Retained	[2]
Sn-NTA	1.16	24.87	83.2	24.04	85% Retained	[3]
Pb-MOF	1.11	24.37	76.2	20.87	86 % Retained	[4]
MOF-525	1.08	23.99	72.1	18.73	76% Retained	[4]
ZnL	1.10	23.72	76.5	20.01	94 % Retained	[5]
In-BTC	1.12	23.55	79.0	20.87	< 80% Retained	[6]
ZIF-8	1.06	23.93	75.6	19.13	93% Retained	[7]

Cu_2O-MIL-53(Al)	1.11	23.89	76.5	20.25	96% Retained	This work

Table S4. Time-resolved PL decay data comparing pristine and CuM-treated perovskite films.

Sample	τ_{ave}/ns	τ_1/ns	A_1	τ_2/ns	A_2
**Perovskite (Pristine)**	8.28	4.09	0.06	13.08	0.04
**Perovskite (CuM 0.075)**	11.45	4.35	0.08	16.62	0.05

$\tau_{avg} = \sum(A_i * \tau_i)^2 / \sum(A_i * \tau_i)$

Table S5. listing of electrochemical impedance spectroscopy (EIS) extracted parameters

Concentration(mg/mL)	R_s (Ω)	R_{rec} (Ω)	C_{rec} nF
Pristine	7.71	351.83	36.2
CuM 0.075	7.99	461.10	70.0

References

1. Lee, C. C.; Chen, C. I.; Liao, Y. T.; Wu, K. C. W.; Chueh, C. C., Enhancing efficiency and stability of photovoltaic cells by using perovskite/Zr-MOF heterojunction including bilayer and hybrid structures. *Advanced Science* **2019,** *6* (5), 1801715.

2. Xiao, B.; Zhang, W.; Xiong, Y.; Huang, Y.; Huang, C.; Qian, Y.; Shen, G.; Basit, A.; Luo, Y.; Li, X., Energy level alignment regulation and carrier management in perovskite solar cells with various bandgaps using tailored metal-organic frameworks. *Advanced Functional Materials* **2025,** *35* (11), 2417293.
3. Zhao, C.; Liu, M.; Liu, X.; Yang, X.; Fan, L.; Wei, M.; Liu, H.; Li, X.; Liu, T.; Li, B., Synergistic Enhancement of Light Harvesting and Interfacial Defect Reduction Using Metal–Organic Frameworks for Efficient and Stable Perovskite Solar Cells. *ACS Sustainable Chemistry & Engineering* **2025,** *13* (13), 5109-5120.
4. Liu, C.-K.; Wu, K.-H.; Lu, Y.-A.; Hsiao, L.-Y.; Lai, K.-W.; Chu, C.-W.; Ho, K.-C., Introducing postmetalation metal–organic framework to control perovskite crystal growth for efficient perovskite solar cells. *ACS applied materials & interfaces* **2021,** *13* (50), 60125-60134.
5. Li, C.; Qiu, J.; Zhu, M.; Cheng, Z.; Zhang, J.; Xiang, S.; Zhang, X.; Zhang, Z., Multifunctional anionic metal-organic frameworks enhancing stability of perovskite solar cells. *Chemical Engineering Journal* **2022,** *433*, 133587.
6. Zhou, X.; Qiu, L.; Fan, R.; Zhang, J.; Hao, S.; Yang, Y., Heterojunction incorporating perovskite and microporous metal–organic framework nanocrystals for efficient and stable solar cells. *Nano-micro letters* **2020,** *12* (1), 80.
7. Li, C.; Guo, S.; Chen, J.; Cheng, Z.; Zhu, M.; Zhang, J.; Xiang, S.; Zhang, Z., Mitigation of vacancy with ammonium salt-trapped ZIF-8 capsules for stable perovskite solar cells through simultaneous compensation and loss inhibition. *Nanoscale advances* **2021,** *3* (12), 3554-3562.
